# Supplementary figures and images for: Diclofenac sensitizes multi-drug resistant Acinetobacter baumannii to colistin
Source: PLoS Pathog. 2024 Nov 21;20(11):e1012705. doi: 10.1371/journal.ppat.1012705 (PMC11620633; doi:10.1371/journal.ppat.1012705)

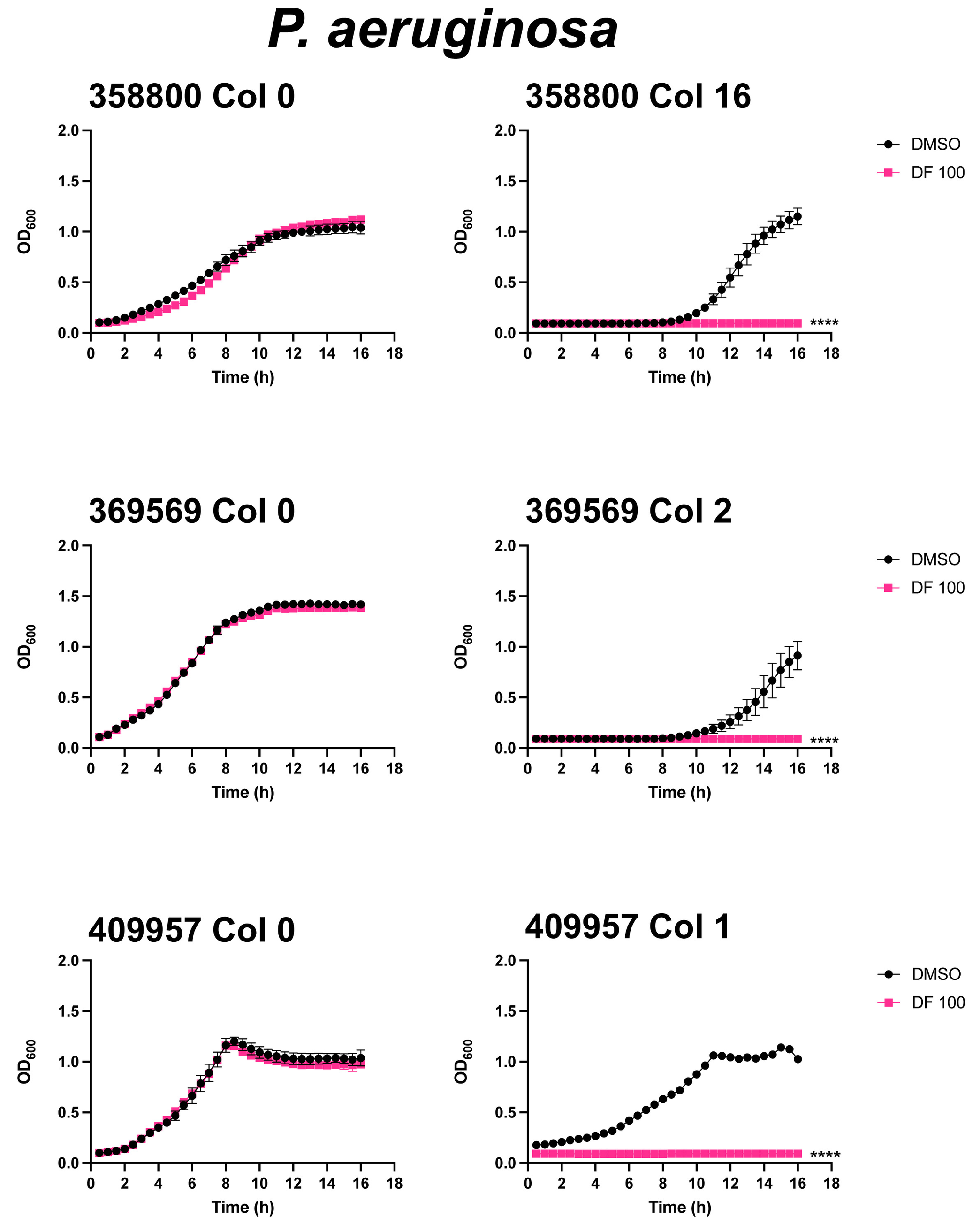

Supplement: S1 Fig — Representative growth curves of P. aeruginosa 358800, 369569, and 409957 strains in LB containing either the solvent control DMSO or 100 μM diclofenac (DF) (left panel) and LB containing 16 μg/ml, 2 μg/ml, or 1 μg/ml colistin respectively in the presence of DMSO or 100 μM diclofenac (right panel). ****P<0.0001, unpaired t tests at 16 h for Col + DF 100 compared to DMSO control. Col (colistin), DF (diclofenac). (TIF) [file ppat.1012705.s001.tif]

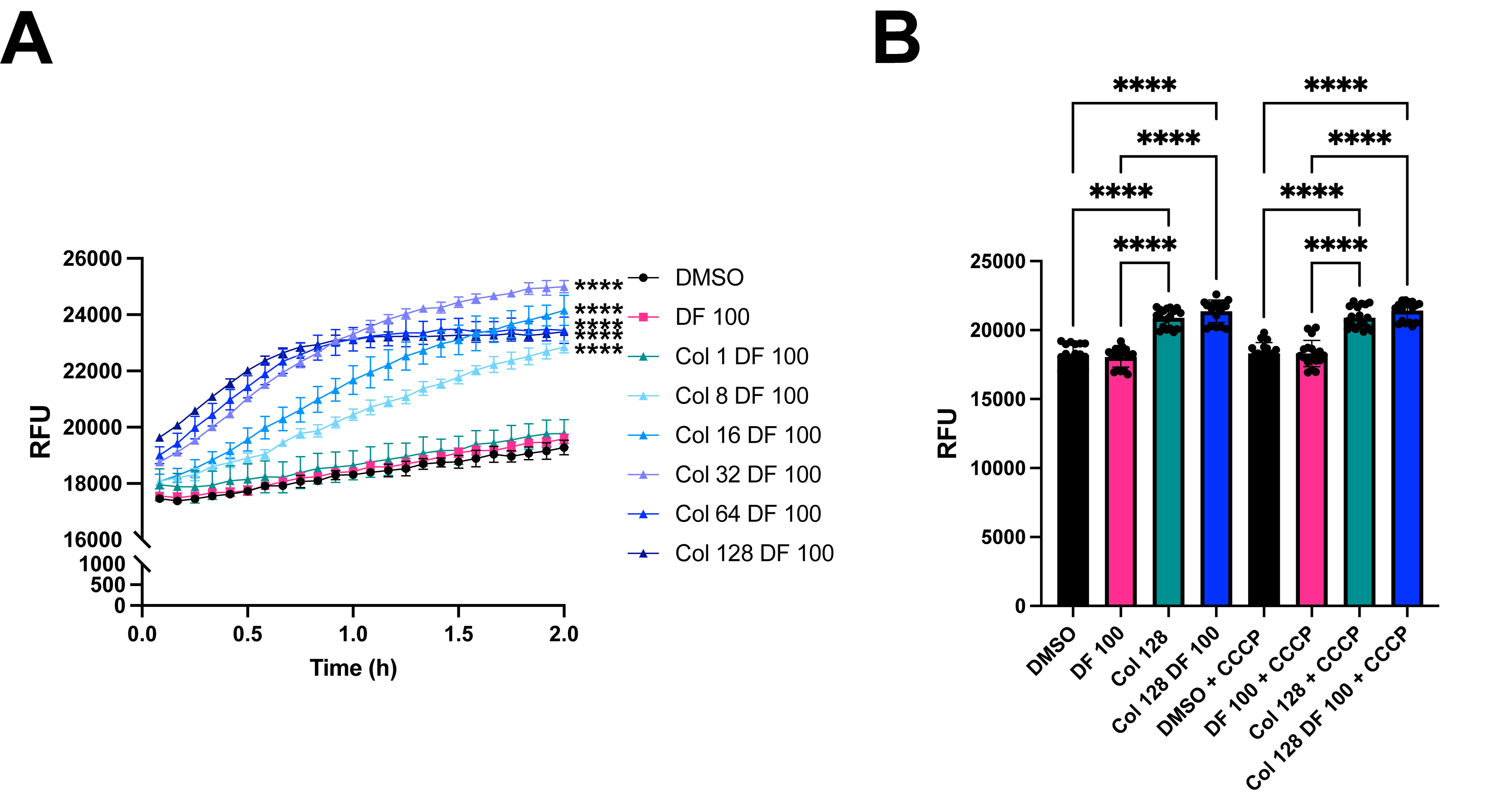

Supplement: S2 Fig — (A) Representative membrane permeability assay measuring uptake of Hoescht 33342 fluorescent dye. Bacterial cells were suspended in PBS supplemented with increasing concentrations of colistin (Col 1 μg/ml, 8 μg/ml, 16 μg/ml, 32 μg/ml, 64 μg/ml, or 128 μg/ml) in combination with the solvent control DMSO or 100 μM DF. Relative fluorescent units (RFU) were monitored over the course of 2 hours. (B) Cell membrane permeabilization assay measuring NPN uptake with or without efflux pump inhibitor CCCP. ****P<0.0001 (One-way ANOVA with Tukey’s test for multiple comparisons, in A results were compared to the control group treated with DMSO). Col (colistin), DF (diclofenac). (TIF) [file ppat.1012705.s002.tif]

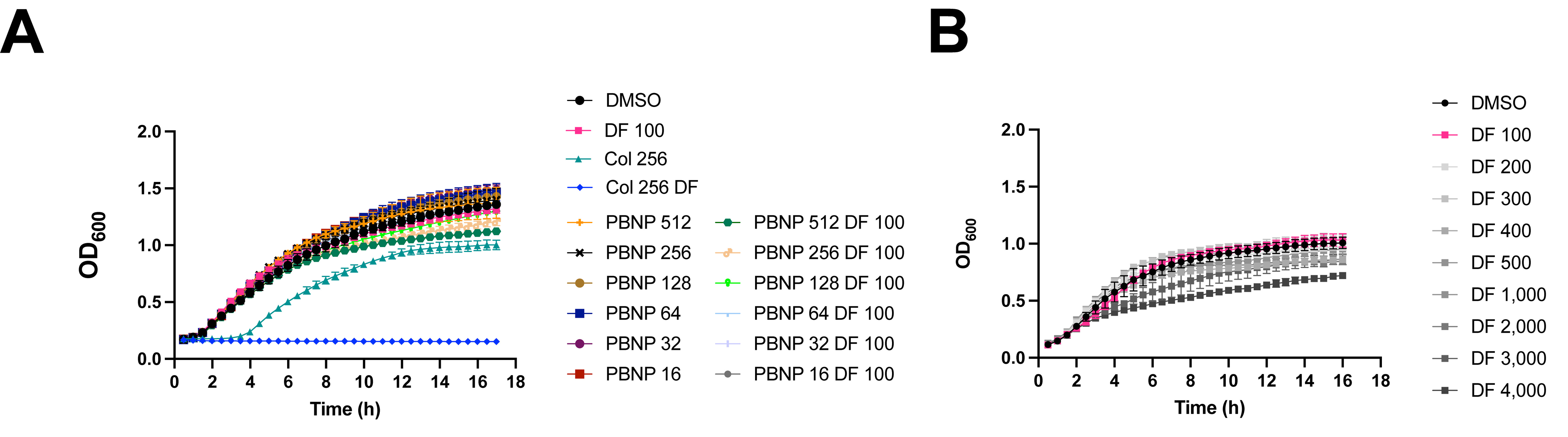

Supplement: S3 Fig — (A) Polymyxin B nanopetide and diclofenac do not affect ARC6851 growth. Representative growth curves of ARC6851 in LB containing increasing concentrations of Polimixin B nanopeptide (PBNP) with or without 100 μM diclofenac. (B) Diclofenac does not affect ARC6851 growth at concentrations up to 4,000 μM. Representative growth curves of ARC6851 in LB containing increasing concentrations of diclofenac (100 μM, 200 μM, 300 μM, 400 μM, 500 μM, 1,000 μM, 2,000 μM, 3,000 μM, or 4000 μM). DF (diclofenac). (TIF) [file ppat.1012705.s003.tif]

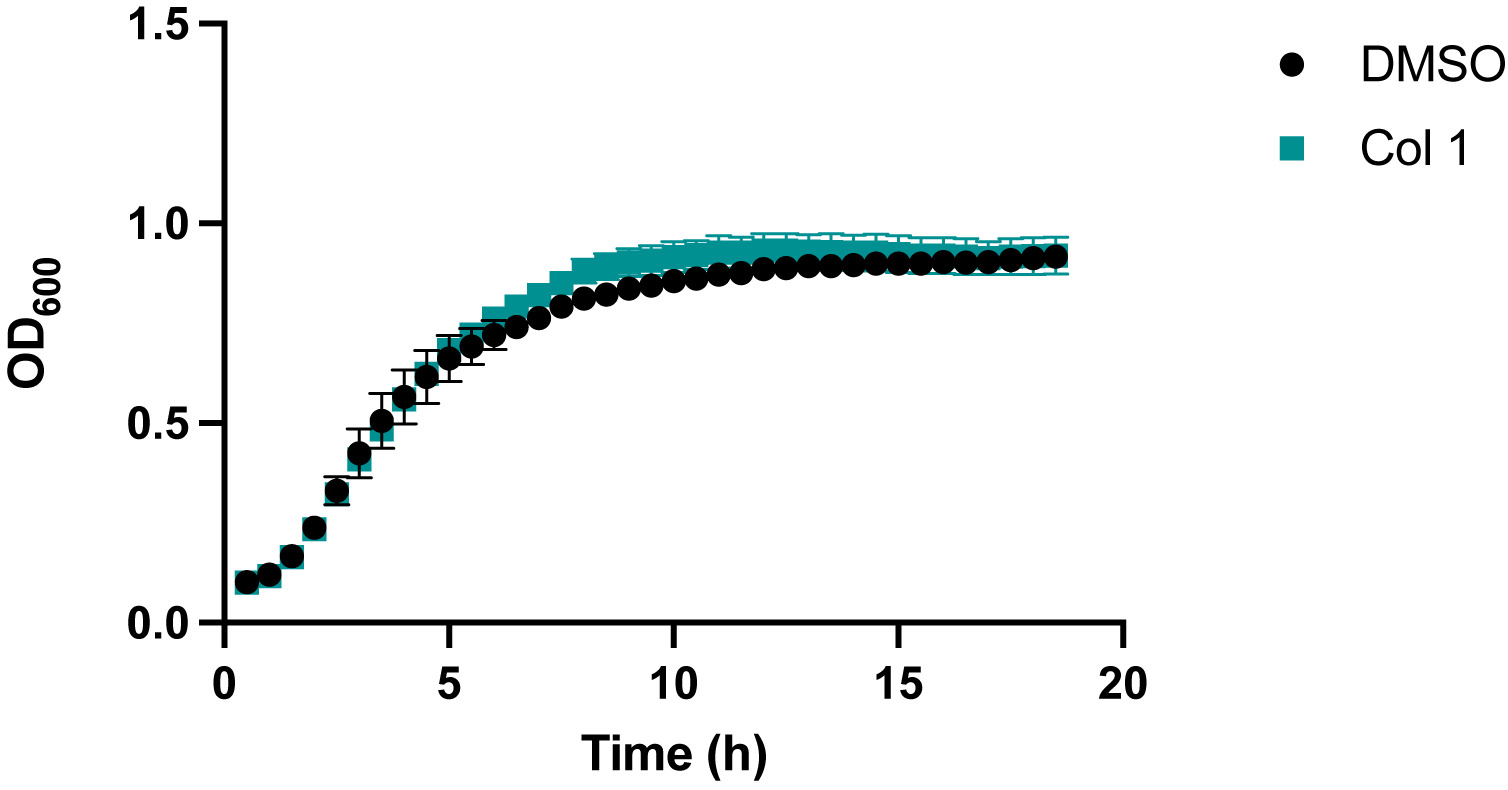

Supplement: S4 Fig — Representative growth curves of ARC6851 in LB containing either the solvent control DMSO or 1 μg/ml colistin. Col (colistin). (TIF) [file ppat.1012705.s004.tif]

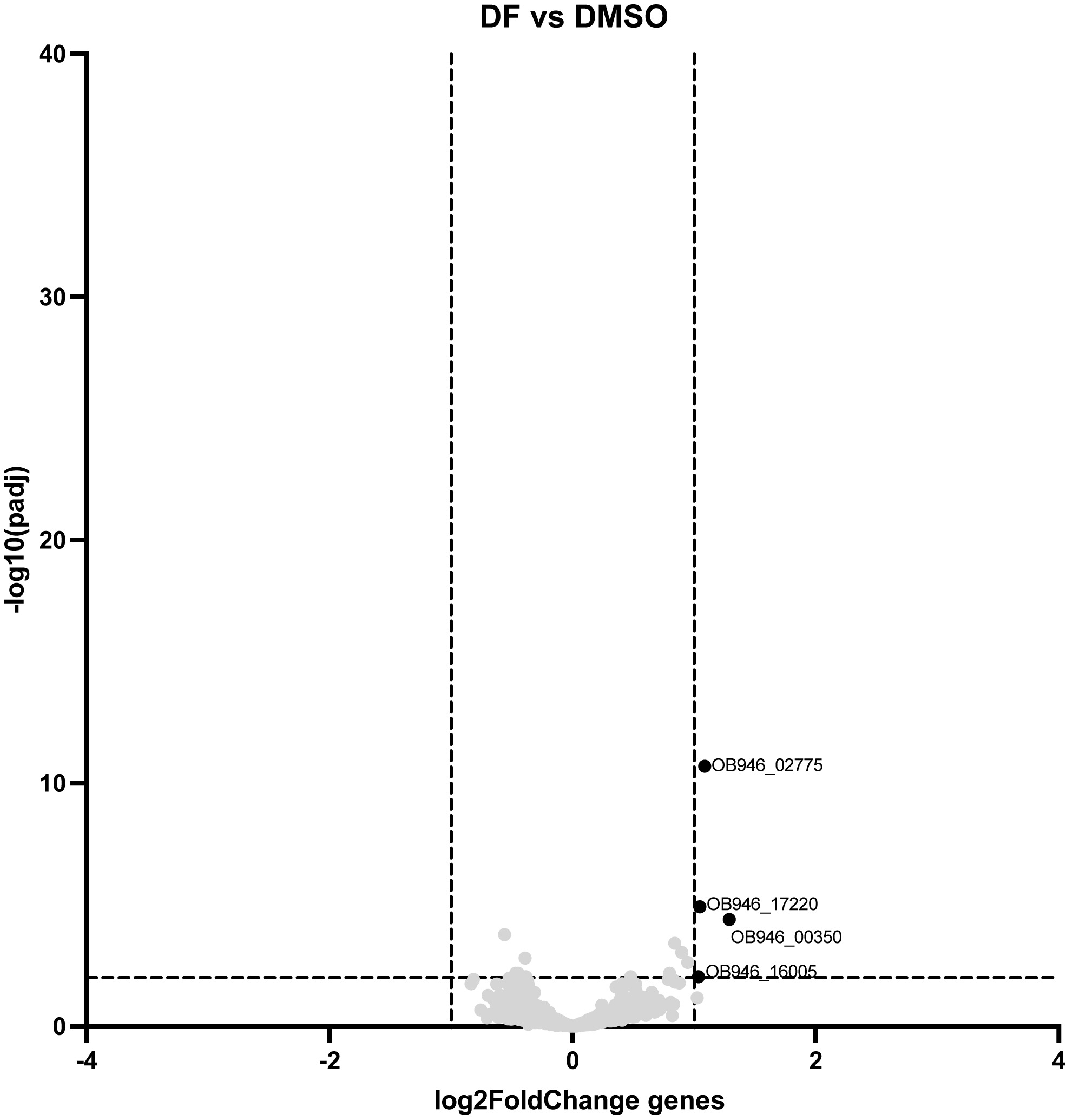

Supplement: S5 Fig — Volcano plot of Supplemental data set showing differentially expressed genes in ARC6851 in diclofenac treatment vs DMSO control. DF (diclofenac). (TIF) [file ppat.1012705.s005.tif]

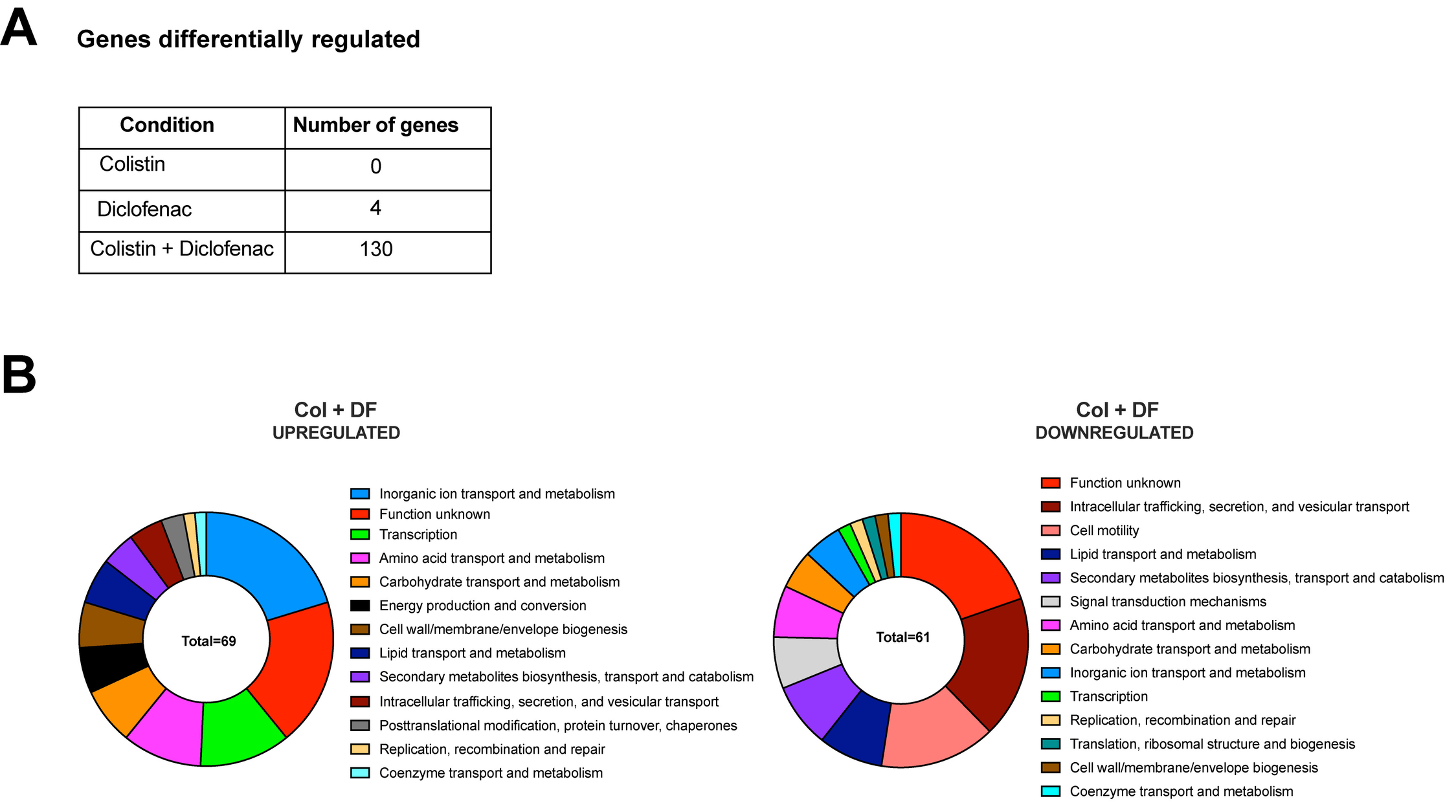

Supplement: S6 Fig — (A) Number of differentially regulated genes in ARC6851 when treated with 1μg/ml colistin, 100 μM diclofenac, or in combination. (B) Donut charts representing the functional classifications of differentially regulated genes of the combination treatment against DMSO. Classifications were determined based on the designated clusters of orthologous groups (COGs) using eggNOG Mapper. Col (colistin), DF (diclofenac). (TIF) [file ppat.1012705.s006.tif]

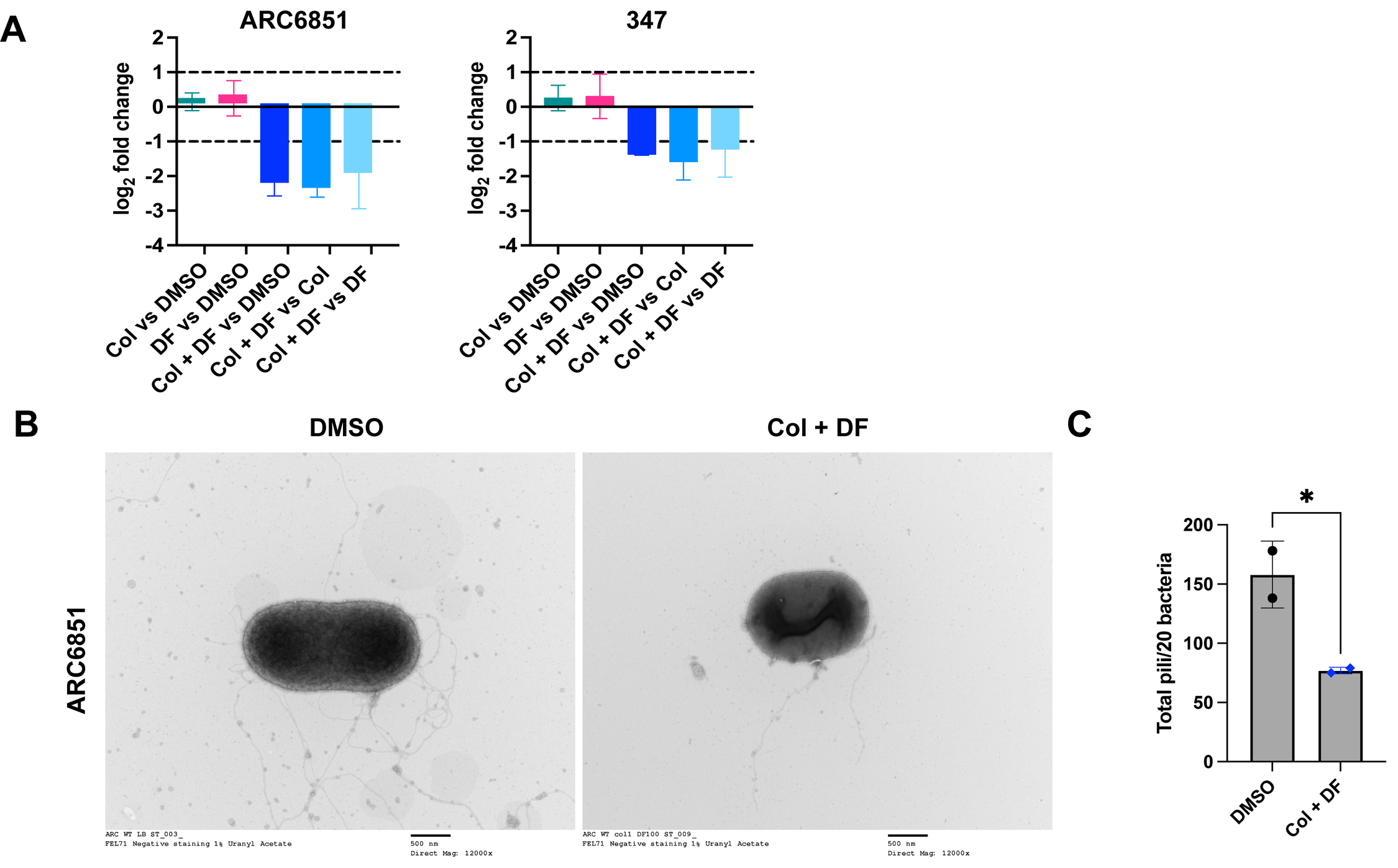

Supplement: S7 Fig — (A) Relative gene expression of pilA in ARC6851 grown in LB + DMSO, LB+ colistin (1 μg/ml) + DMSO, LB + diclofenac (100 μM), or LB + colistin (1 μg/ml) + diclofenac (100 μM) as determined by qRT-PCR. Dotted lines represent 2-fold change. (B) Transmission electron microscopy of ARC6851 WT in LB plus DMSO and LB plus colistin (1 μg/ml) plus diclofenac (100 μM). Scale bar of 500 nm is shown. (C) Quantification of type IV pili in ARC6851 WT was performed in 20 bacterial cells. Col (colistin), DF (diclofenac). Unpaired t tests *P < 0.05. (TIF) [file ppat.1012705.s007.tif]

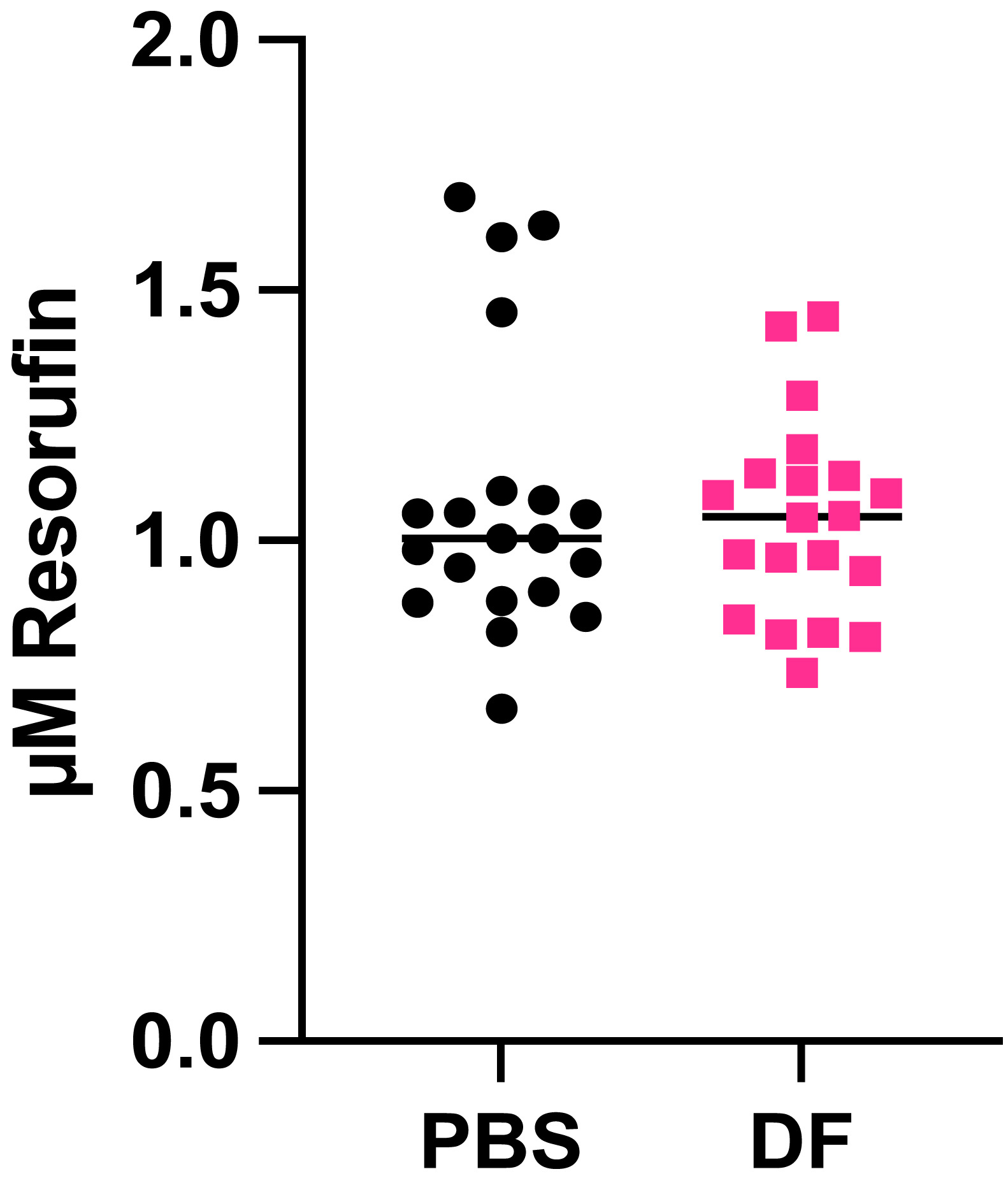

Supplement: S8 Fig — Lung lysates containing protease inhibitors were used to determine COX-2 activities using a COX fluorescent activity assay kit. Resorufin fluorescence can be analyzed with an excitation wavelength between 530–540 nm and an emission wavelength between 585–595 nm. DF (diclofenac). (TIF) [file ppat.1012705.s008.tif]

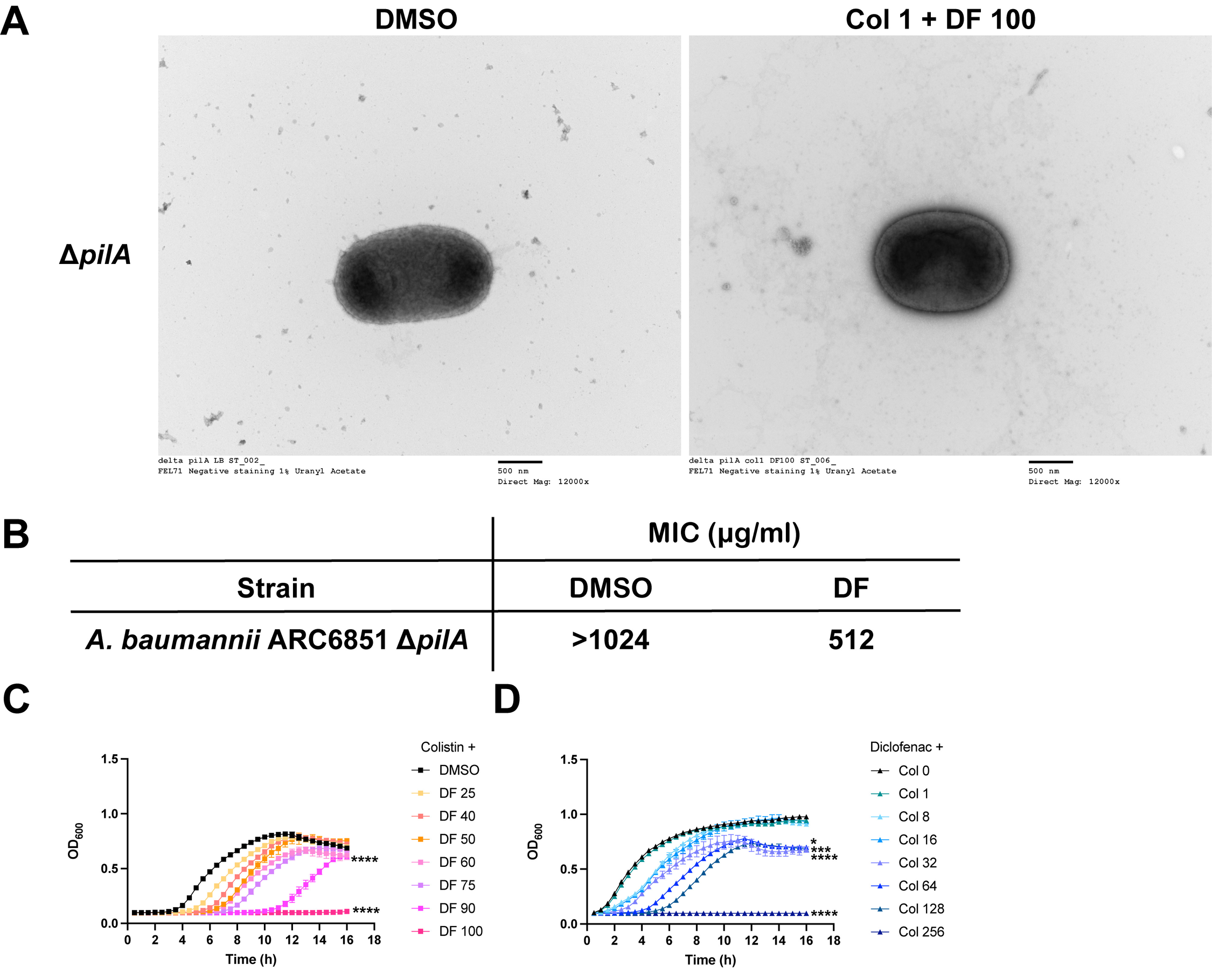

Supplement: S9 Fig — (A) Transmission electron microscopy of ARC6851 ΔpilA in LB plus DMSO and LB plus colistin (1 μg/ml) plus diclofenac (100 μM). Scale bar of 500 nm is shown. (B) ΔpilA was screened for changes in MICs to colistin in combination with the solvent control DMSO and 100 μM diclofenac using a 2-fold broth dilution method. MIC was determined as <10% growth compared to a non-treated culture. (C) Representative growth curves of ARC6851 ΔpilA in LB containing 256 μg/ml colistin with increasing concentrations of diclofenac (25 μM, 40 μM, 50 μM, 60 μM, 75 μM, 90 μM, or 100 μM). (D) Representative growth curves of ARC6851 ΔpilA in LB containing 100 μM diclofenac with increasing concentrations of colistin (1 μg/ml, 8 μg/ml, 16 μg/ml, 32 μg/ml, 64 μg/ml, 128 μg/ml, or 256 μg/ml). *P<0.05, ***P<0.001, ****P<0.0001. (TIF) [file ppat.1012705.s009.tif]

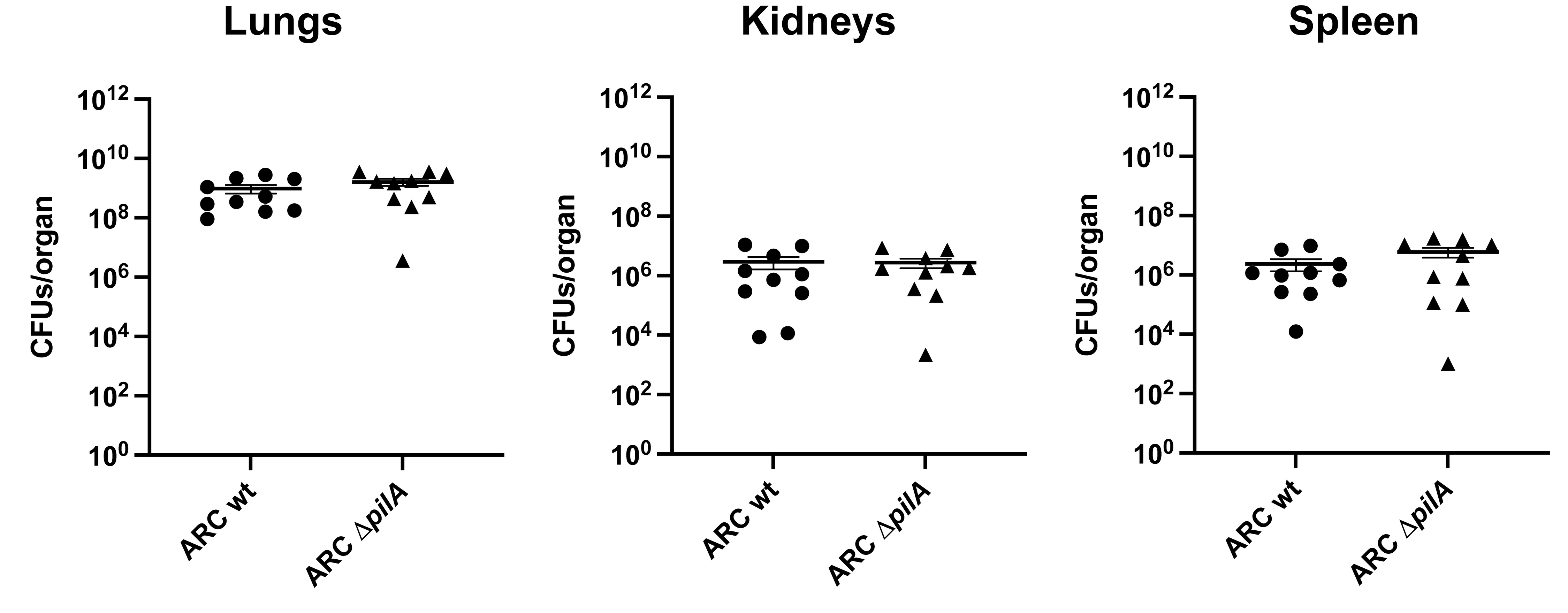

Supplement: S10 Fig — C57BL/6 mice were infected with ~5 × 10^7^ CFU of mid-exponential ARC6851 WT or ΔpilA. At 24 h post-infection, the lungs, kidneys, and spleens were harvested, and the bacterial load present in each tissue was determined with serial dilutions. Each symbol represents an individual mouse, and the horizontal bar represents the Mean with SEM. Data collected from two independent experiments. *P < 0.05, **P < 0.01, Kruskal-Wallis test. (TIF) [file ppat.1012705.s010.tif]

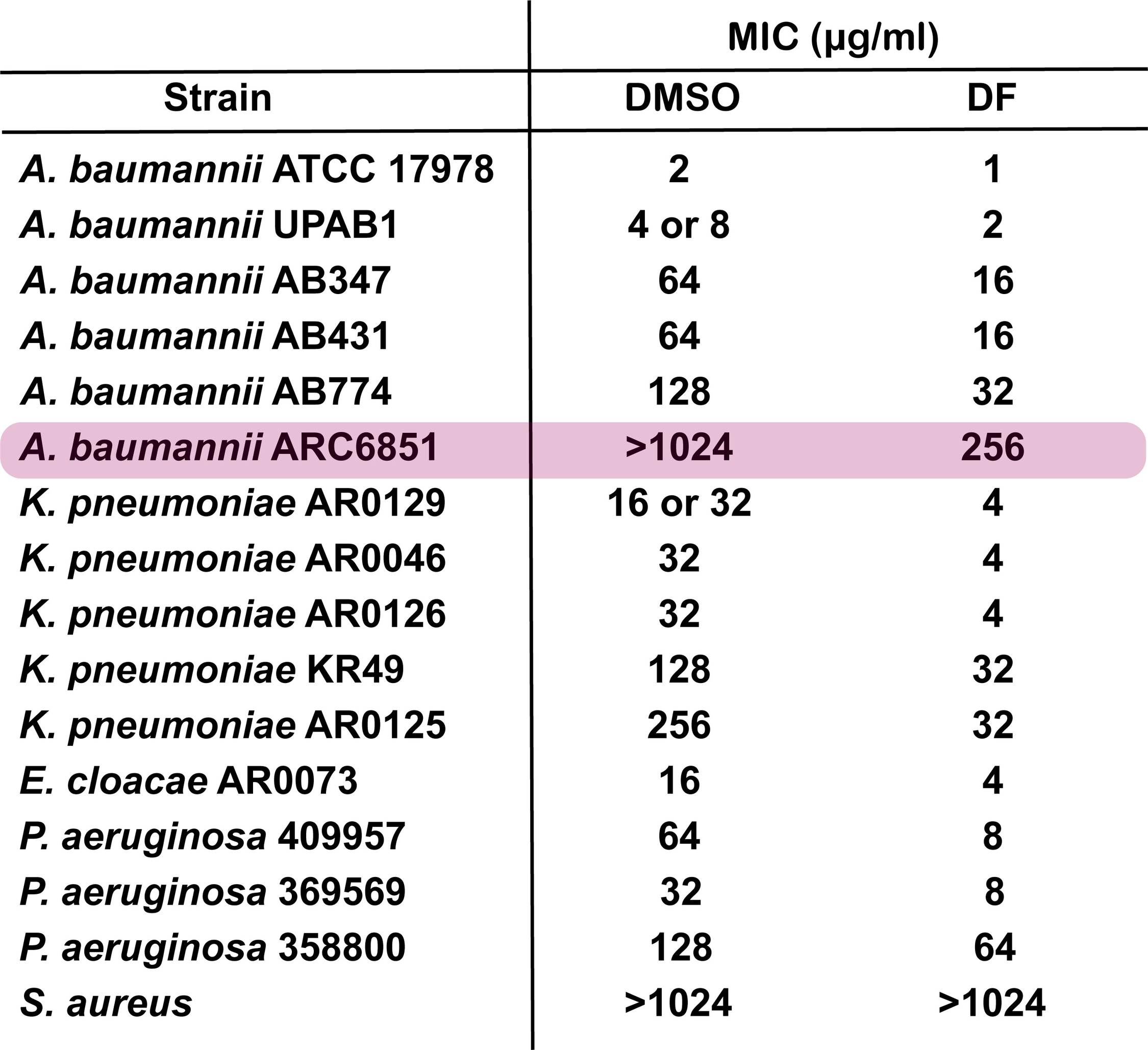

Supplement: S1 Table — Gram-negative and Gram-positive bacteria were screened for changes in MICs to colistin in combination with the solvent control DMSO or 100 μM diclofenac using a 2-fold broth dilution method. MIC was determined as <10% growth compared to a non-treated culture. DF (diclofenac). (TIF) [file ppat.1012705.s011.tif]

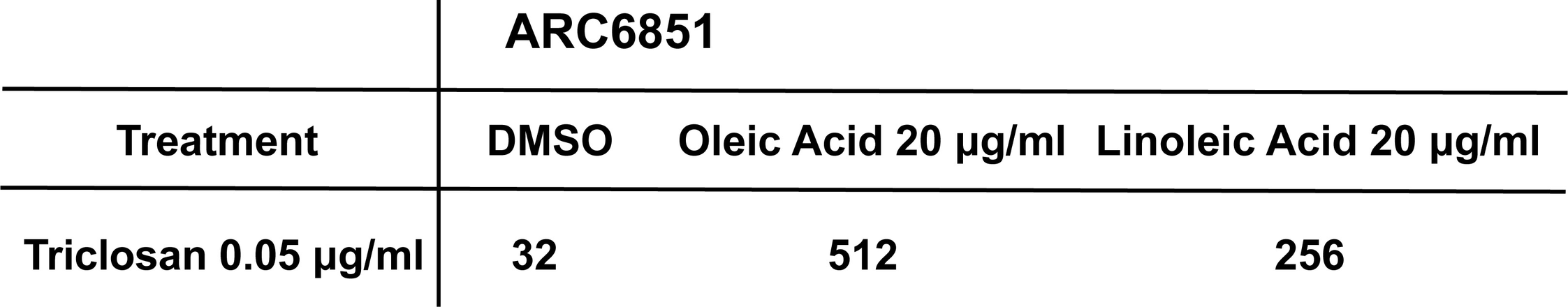

Supplement: S2 Table — ARC6851 was screened for changes in MICs to triclosan in combination with either the solvent control DMSO or 20 μg/ml of oleic acid and linoleic acid, using a 2-fold broth dilution method. MIC was determined as <10% growth compared to a non-treated culture. (TIF) [file ppat.1012705.s012.tif]

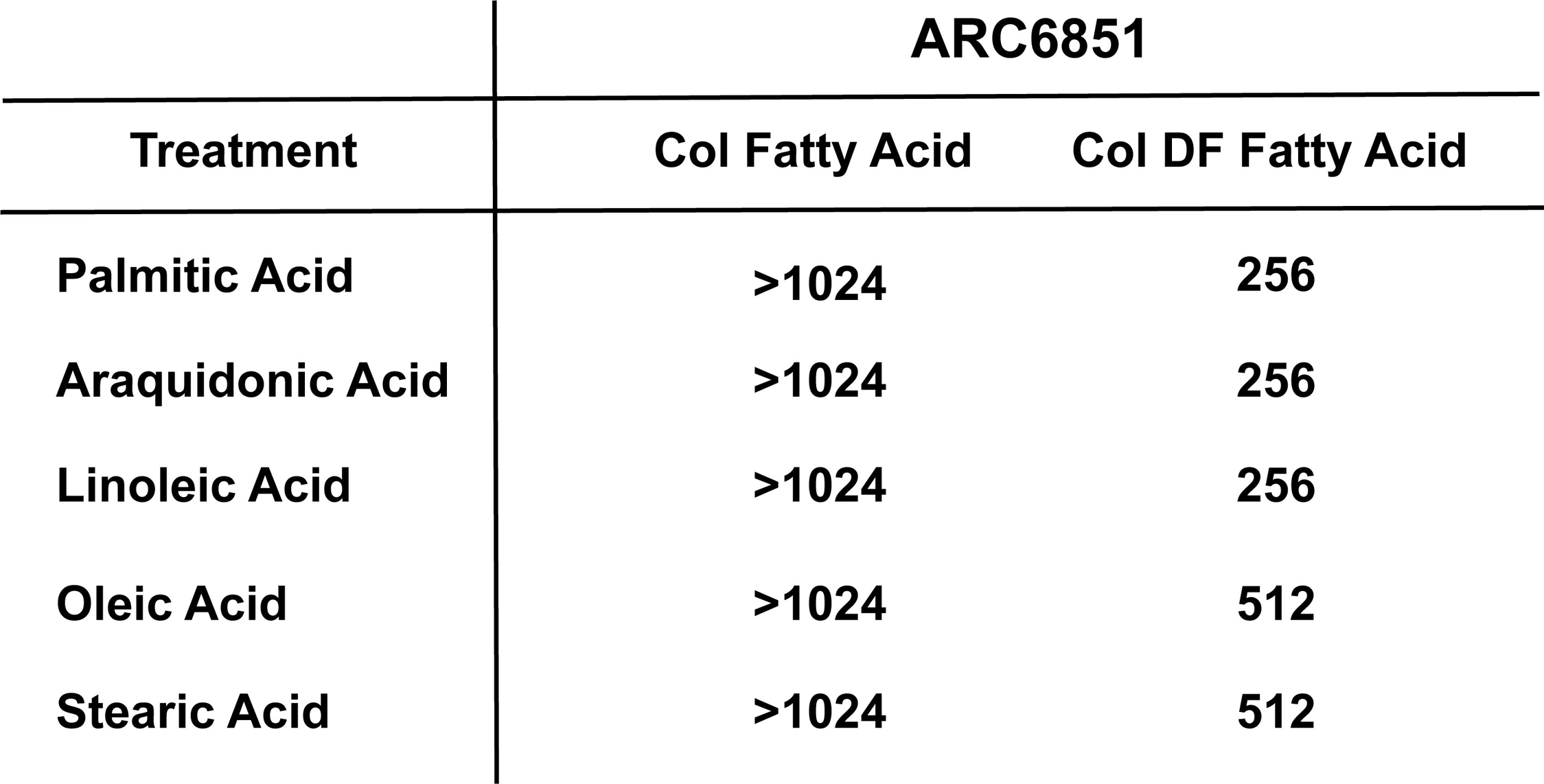

Supplement: S3 Table — ARC6851 was screened for changes in MICs to colistin in combination with either the solvent control DMSO or 20 μg/ml of palmitic acid, araquidonic acid, linoleic acid, oleic acid, and stearic acid using a 2-fold broth dilution method. MIC was determined as <10% growth compared to a non-treated culture. Col (colistin), DF (diclofenac). (TIF) [file ppat.1012705.s013.tif]

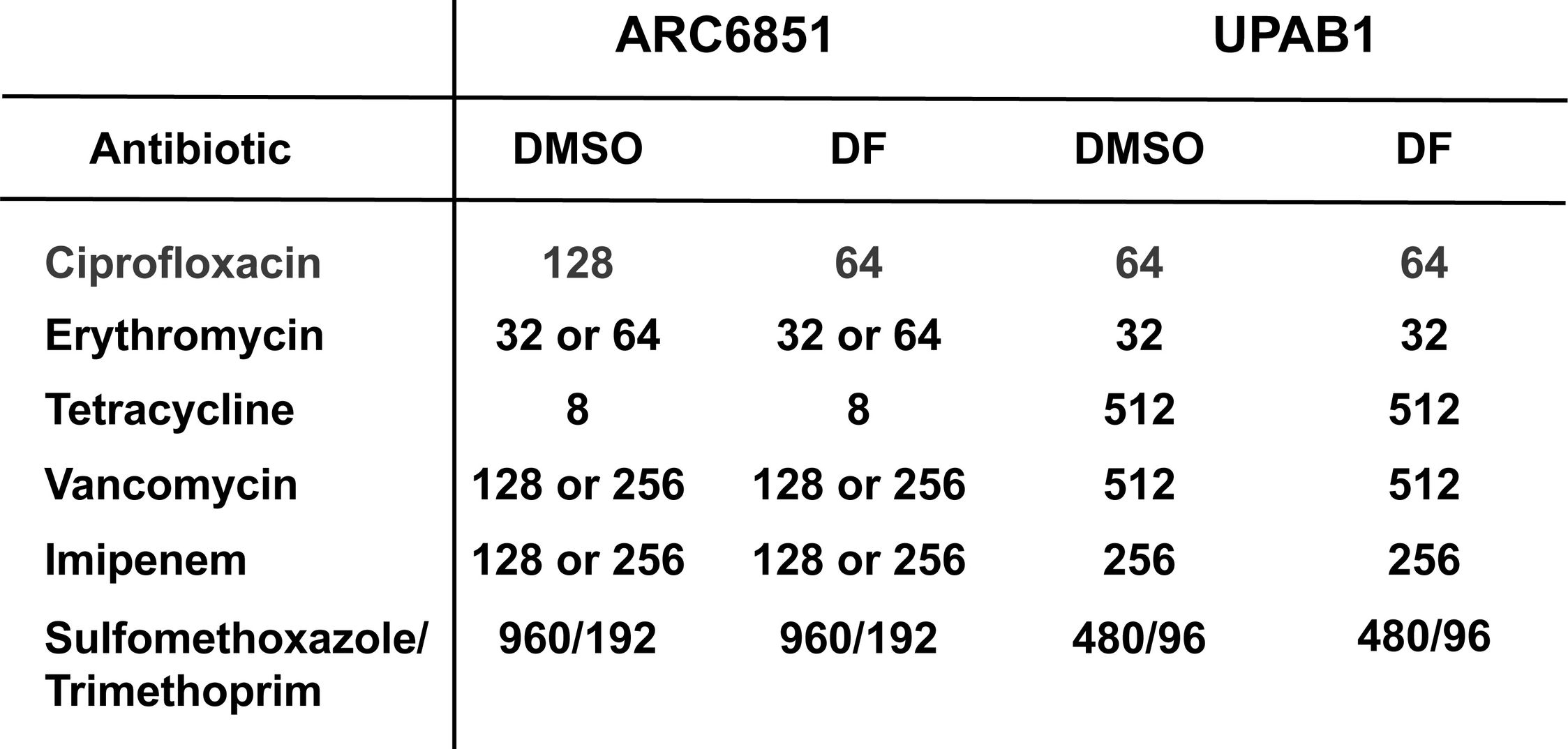

Supplement: S4 Table — ARC6851 and UPAB1 were screened for changes in MICs to ciprofloxacin, erythromycin, tetracycline, vancomycin, imipenem, or sulfomethoxazole/trimethoprim in combination with the solvent control DMSO or 100 μM diclofenac using a 2-fold broth dilution method. MIC was determined as <10% growth compared to a non-treated culture. DF (diclofenac). (TIF) [file ppat.1012705.s014.tif]
