# Supplementary material for: Diclofenac sensitizes multi-drug resistant Acinetobacter baumannii to colistin
Source: PLoS Pathog. 2024 Nov 21;20(11):e1012705. doi: 10.1371/journal.ppat.1012705 (PMC11620633; doi:10.1371/journal.ppat.1012705)
Supplement: S5 Table — (DOCX) [file ppat.1012705.s015.docx]

**Table S5: Upregulated genes in ARC6851 in diclofenac treatment vs DMSO.**

| **Accession** | **Annotated gene** | **Fold change^a^** |
| --- | --- | --- |
| OB946_00350 | fahA | 2.44 |
| OB946_02775 | Acyl-CoA dehydrogenase C-terminal domain-containing protein | 2.12 |
| OB946_17220 | fadB | 2.06 |
| OB946_16005 | Lipid transport and metabolism | 2.05 |

a| Fold change cutoff: 2-fold *P* value < 0.01. Differential expression was calculated with DESeq2.
