# Supplementary material for: Diclofenac sensitizes multi-drug resistant Acinetobacter baumannii to colistin
Source: PLoS Pathog. 2024 Nov 21;20(11):e1012705. doi: 10.1371/journal.ppat.1012705 (PMC11620633; doi:10.1371/journal.ppat.1012705)
Supplement: S6 Table — (DOCX) [file ppat.1012705.s016.docx]

**Table S6: Differentially expressed genes in ARC6851 in colistin + diclofenac treatment vs DMSO.**

| **Accession** | **Annotated gene** | **Fold change^a^** |
| --- | --- | --- |
| **OB946_12785** | **NAD(P)/FAD-dependent oxidoreductase** | **11.37** |
| **OB946_12790** | **SDR family oxidoreductase** | **10.61** |
| **OB946_00885** | **FMN reductase** | **6.11** |
| **OB946_18865** | **RcnB family protein** | **5.64** |
| OB946_04015 | TetR/AcrR family transcriptional regulator | 5.56 |
| OB946_01760 | hypothetical protein | 5.52 |
| OB946_09560 | hypothetical protein | 4.99 |
| OB946_15220 | TetR/AcrR family transcriptional regulator | 4.68 |
| **OB946_02760** | **RcnB family protein** | **4.65** |
| OB946_12795 | alpha/beta hydrolase | 4.29 |
| OB946_02780 | hypothetical protein | 4.11 |
| **OB946_15490** | **MFS transporter** | **4.00** |
| **OB946_10710** | **SfnB family sulfur acquisition oxidoreductase** | **3.65** |
| OB946_19645 |  | 3.50 |
| OB946_09895 | sulfite exporter TauE/SafE family protein | 3.48 |
| **OB946_18890** | **aliphatic sulfonate ABC transporter permease SsuC** | **3.31** |
| **OB946_00880** | **dimethyl sulfone monooxygenase SfnG** | **3.18** |
| **OB946_18885** | **FMNH2-dependent alkanesulfonate monooxygenase** | **3.11** |
| OB946_09155 | hypothetical protein | 3.11 |
| **OB946_18880** | **sulfonate ABC transporter substrate-binding protein** | **3.11** |
| OB946_08735 | MBL fold metallo-hydrolase | 3.01 |
| **OB946_02885** | **peroxiredoxin** | **2.91** |
| **OB946_10965** | **monooxygenase** | **2.88** |
| **OB946_18860** | **RcnB family protein** | **2.87** |
| OB946_09715 | lipoyl synthase | 2.81 |
| OB946_10655 | hypothetical protein | 2.79 |
| OB946_17300 | hypothetical protein | 2.78 |
| OB946_08740 | TetR family transcriptional regulator | 2.77 |
| OB946_18980 | hypothetical protein | 2.73 |
| **OB946_16170** | **MacA family efflux pump subunit** | **2.70** |
| **OB946_09580** | **MFS transporter** | **2.67** |
| OB946_00905 | DUF485 domain-containing protein | 2.61 |
| OB946_17670 | signal peptidase II | 2.55 |
| OB946_15495 | substrate-binding domain-containing protein | 2.54 |
| OB946_07115 | hypothetical protein | 2.54 |
| OB946_01830 | TIGR03915 family putative DNA repair protein | 2.50 |
| **OB946_10945** | **taurine ABC transporter substrate-binding protein** | **2.50** |
| OB946_18985 | hypothetical protein | 2.47 |
| **OB946_10720** | **LLM class flavin-dependent oxidoreductase** | **2.44** |
| OB946_18255 | hypothetical protein | 2.42 |
| OB946_19015 | M57 family metalloprotease | 2.42 |
| OB946_10755 | Crp/Fnr family transcriptional regulator | 2.40 |
| **OB946_18240** | **MFS transporter** | **2.39** |
| **OB946_03935** | **multidrug efflux RND transporter permease subunit** | **2.39** |
| OB946_14280 | phage tail protein | 2.39 |
| OB946_10925 | aspartate/glutamate racemase family protein | 2.39 |
| OB946_18645 | GntR family transcriptional regulator | 2.38 |
| **OB946_13205** | **cation diffusion facilitator family transporter** | **2.34** |
| OB946_02765 | phosphate-starvation-inducible PsiE family protein | 2.34 |
| OB946_13200 | LysE/ArgO family amino acid transporter | 2.32 |
| **OB946_15225** | **MFS transporter** | **2.22** |
| OB946_19640 |  | 2.21 |
| OB946_14925 | lipoprotein insertase outer membrane protein LolB | 2.20 |
| OB946_09705 | alpha-ketoacid dehydrogenase subunit beta | 2.20 |
| OB946_15450 | DUF1852 domain-containing protein | 2.17 |
| **OB946_09500** | **MFS transporter** | **2.16** |
| OB946_02270 | DUF3108 domain-containing protein | 2.16 |
| OB946_17295 | YMGG-like glycine zipper-containing protein | 2.15 |
| **OB946_04415** | **sulfate ABC transporter permease subunit CysT** | **2.13** |
| OB946_13800 | BCCT family transporter | 2.13 |
| OB946_18370 | PadR family transcriptional regulator | 2.13 |
| **OB946_10940** | **ATP-binding cassette domain-containing protein** | **2.12** |
| **OB946_04885** | **phosphate ABC transporter permease subunit PstC** | **2.11** |
| OB946_01140 | hypothetical protein | 2.11 |
| OB946_16770 | LysR family transcriptional regulator | 2.11 |
| OB946_16295 | class I SAM-dependent methyltransferase | 2.10 |
| **OB946_09460** | **anthranilate 1,2-dioxygenase small subunit** | **2.09** |
| OB946_19635 | DUF3861 domain-containing protein | 2.08 |
| OB946_18365 | DUF3861 domain-containing protein | 2.08 |
| OB946_10995 | LysR family transcriptional regulator | 2.08 |
| **OB946_00330** | **4-hydroxyphenylpyruvate dioxygenase** | **2.05** |
| OB946_03965 | transglycosylase SLT domain-containing protein | 2.05 |
| **OB946_04410** | **sulfate ABC transporter permease subunit CysW** | **2.05** |
| OB946_18195 | hypothetical protein | 2.04 |
| OB946_08485 | DUF6438 domain-containing protein | -2.04 |
| OB946_17110 | type II secretion system F family protein | -2.04 |
| OB946_15850 | hypothetical protein | -2.08 |
| OB946_01575 | VWA domain-containing protein | -2.08 |
| OB946_08640 | catechol 1,2-dioxygenase | -2.08 |
| OB946_04395 | ornithine uptake porin CarO type 1 | -2.08 |
| OB946_08695 | alpha/beta hydrolase | -2.13 |
| OB946_17055 | Na+/H+ antiporter subunit G | -2.13 |
| OB946_04995 | EAL domain-containing protein | -2.17 |
| OB946_09245 | hypothetical protein | -2.17 |
| OB946_08230 | cyd operon YbgE family protein | -2.17 |
| OB946_09660 | FAD/NAD(P)-binding oxidoreductase | -2.17 |
| OB946_09670 | MBL fold metallo-hydrolase | -2.17 |
| **OB946_01455** | **type IV pilus secretin PilQ family protein** | **-2.22** |
| OB946_03135 | methyl-accepting chemotaxis protein | -2.27 |
| **OB946_11650** | **PaaI family thioesterase** | -2.27 |
| OB946_11195 | LysE family translocator | **-2.27** |
| OB946_08645 | muconolactone Delta-isomerase | -2.33 |
| OB946_07615 | benzoate/H(+) symporter BenE family transporter | -2.33 |
| OB946_19040 | DMT family protein | -2.33 |
| **OB946_10590** | **fimbrial protein** | **-2.33** |
| OB946_05255 | DUF3015 family protein | -2.33 |
| OB946_15250 | mechanosensitive ion channel | -2.38 |
| OB946_03145 | hypothetical protein | -2.44 |
| OB946_18220 | DNA-processing protein DprA | -2.44 |
| **OB946_01560** | **type IV pilus modification protein PilV** | **-2.44** |
| OB946_04335 | multidrug effflux MFS transporter | -2.44 |
| OB946_12235 | DUF4882 family protein | -2.50 |
| OB946_04785 | type I-F CRISPR-associated endoribonuclease Cas6/Csy4 | -2.50 |
| **OB946_11660** | **PaaX** | **-2.50** |
| **OB946_01565** | **PilW family protein** | **-2.63** |
| OB946_15885 | 50S ribosomal protein L35 | -2.63 |
| OB946_10625 | hypothetical protein | -2.70 |
| **OB946_01450** | **pilus assembly protein PilP** | **-2.78** |
| **OB946_11685** | **enoyl-CoA hydratase-related protein** | **-2.78** |
| OB946_09310 | hypothetical protein | -2.78 |
| OB946_00200 | MFS transporter | -2.78 |
| OB946_04780 | type I-F CRISPR-associated protein Csy3 | -2.86 |
| OB946_08785 | hydrolase | -2.86 |
| **OB946_01555** | **GspH/FimT family pseudopilin** | **-2.86** |
| **OB946_11680** | **2-(1,2-epoxy-1,2-dihydrophenyl)acetyl-CoA** | **-2.86** |
| OB946_03140 | isomerase PaaG | -2.94 |
| **OB946_11665** | **phenylacetate--CoA ligase PaaK** | **-2.94** |
| OB946_13975 | hypothetical protein | -3.03 |
| OB946_01570 | hypothetical protein | -3.03 |
| OB946_03165 | entericidin A/B family lipoprotein | -3.13 |
| OB946_09665 | protein tyrosine phosphatase family protein | -3.33 |
| OB946_09285 | hypothetical protein | -3.57 |
| OB946_09225 | hypothetical protein | -3.57 |
| OB946_15000 | peptidoglycan-binding protein LysM | -3.70 |
| **OB946_11655** | **DapH/DapD/GlmU-related protein** | **-3.85** |
| OB946_09280 | hypothetical protein | -3.85 |
| OB946_07585 | universal stress protein | -4.00 |
| **OB946_11675** | **3-hydroxyacyl-CoA dehydrogenase** | **-4.00** |
| **OB946_14630** | **PilT/PilU family type 4a pilus ATPase** | **-4.00** |
| OB946_10620 | OB946_10620 | -4.17 |
| **OB946_11670** | **3-oxoadipyl-CoA thiolase** | **-5.26** |
| OB946_09205 | hypothetical protein | -5.26 |
| **OB946_01505** | **pilin** | **-9.09** |
| OB946_09275 | major capsid protein | -12.50 |
| OB946_09200 | major capsid protein | -12.50 |

a| Fold change cutoff: 2-fold *P* value < 0.01. Differential expression was calculated with DESeq2.
