# Supplementary material for: Diclofenac sensitizes multi-drug resistant Acinetobacter baumannii to colistin
Source: PLoS Pathog. 2024 Nov 21;20(11):e1012705. doi: 10.1371/journal.ppat.1012705 (PMC11620633; doi:10.1371/journal.ppat.1012705)
Supplement: S7 Table — (DOCX) [file ppat.1012705.s017.docx]

**Table S7: Differentially expressed genes in ARC6851 in colistin + diclofenac treatment vs colistin.**

| **Accession** | **Annotated gene** | **Fold change^a^** |
| --- | --- | --- |
| **OB946_12790** | **SDR family oxidoreductase** | **11.90** |
| **OB946_15490** | MFS transporter | 10.24 |
| **OB946_12785** | **NAD(P)/FAD-dependent oxidoreductase** | **9.67** |
| **OB946_00885** | FMN reductase | 8.90 |
| **OB946_01760** | hypothetical protein | 7.10 |
| **OB946_18875** | sulfonate ABC transporter substrate-binding protein | 6.53 |
| **OB946_18475** | LuxR family transcriptional regulator AbaR | 6.03 |
| **OB946_19460** |  | 5.84 |
| **OB946_12795** | alpha/beta hydrolase | 5.78 |
| **OB946_17865** | site-specific integrase | 5.50 |
| **OB946_06760** | hypothetical protein | 5.34 |
| **OB946_12665** | hypothetical protein | 5.30 |
| **OB946_15220** | TetR/AcrR family transcriptional regulator | 5.28 |
| **OB946_01260** | hypothetical protein | 5.24 |
| **OB946_18980** | hypothetical protein | 5.10 |
| **OB946_08890** | DMT family transporter | 4.96 |
| **OB946_12935** | hypothetical protein | 4.92 |
| **OB946_19570** |  | 4.87 |
| **OB946_00905** | DUF485 domain-containing protein | 4.82 |
| **OB946_18885** | **FMNH2-dependent alkanesulfonate monooxygenase** | **4.73** |
| **OB946_08510** | LysR family transcriptional regulator | 4.61 |
| **OB946_09895** | sulfite exporter TauE/SafE family protein | 4.58 |
| **OB946_00715** | DUF1737 domain-containing protein | 4.57 |
| **OB946_17925** | phage virion morphogenesis protein | 4.30 |
| **OB946_18985** | hypothetical protein | 4.16 |
| **OB946_01140** | hypothetical protein | 4.05 |
| **OB946_01200** | DMT family transporter | 3.81 |
| **OB946_00880** | **dimethyl sulfone monooxygenase SfnG** | **3.80** |
| **OB946_13255** | hypothetical protein | 3.74 |
| **OB946_02760** | **RcnB family protein** | **3.73** |
| **OB946_02885** | **peroxiredoxin** | **3.73** |
| **OB946_18865** | **RcnB family protein** | **3.70** |
| **OB946_10925** | aspartate/glutamate racemase family protein | 3.70 |
| **OB946_01155** | hypothetical protein | 3.70 |
| **OB946_08055** | Lrp/AsnC family transcriptional regulator GigD | 3.68 |
| **OB946_17775** | ogr/Delta-like zinc finger family protein | 3.67 |
| **OB946_11820** | ankyrin repeat domain-containing protein | 3.66 |
| **OB946_13190** | hypothetical protein | 3.65 |
| **OB946_15450** | DUF1852 domain-containing protein | 3.64 |
| **OB946_08435** | 3-carboxy-cis%2Ccis-muconate cycloisomerase | 3.61 |
| **OB946_16795** | acyl-CoA dehydrogenase family protein | 3.60 |
| **OB946_05125** | hypothetical protein | 3.56 |
| **OB946_14190** | hypothetical protein | 3.56 |
| **OB946_05795** | OmpW family outer membrane protein | 3.53 |
| **OB946_04425** | sulfate ABC transporter substrate-binding protein | 3.50 |
| **OB946_08865** | SLC13 family permease | 3.50 |
| **OB946_13205** | cation diffusion facilitator family transporter | 3.45 |
| **OB946_09560** | hypothetical protein | 3.41 |
| **OB946_16570** | winged helix-turn-helix transcriptional regulator | 3.39 |
| **OB946_08740** | TetR family transcriptional regulator | 3.38 |
| **OB946_11725** | APC family permease | 3.38 |
| **OB946_14340** | hypothetical protein | 3.35 |
| **OB946_18645** | **GntR family transcriptional regulator** | **3.31** |
| **OB946_19480** |  | 3.30 |
| **OB946_05030** | LysR substrate-binding domain-containing protein | 3.30 |
| **OB946_12395** | cold-shock protein | 3.30 |
| **OB946_15495** | substrate-binding domain-containing protein | 3.13 |
| **OB946_17165** | DUF1656 domain-containing protein | 3.12 |
| **OB946_01485** | metal-dependent hydrolase | 3.10 |
| **OB946_01170** | AraC family transcriptional regulator | 3.09 |
| **OB946_18370** | PadR family transcriptional regulator | 3.08 |
| **OB946_04015** | TetR/AcrR family transcriptional regulator | 3.06 |
| **OB946_00650** | 2-oxo-4-hydroxy-4-carboxy-5-ureidoimidazoline decarboxylase | 3.06 |
| **OB946_07120** | glycine zipper domain-containing protein | 3.06 |
| **OB946_17300** | hypothetical protein | 3.04 |
| **OB946_19015** | M57 family metalloprotease | 3.04 |
| **OB946_08465** | type I 3-dehydroquinate dehydratase | 3.02 |
| **OB946_14445** | hypothetical protein | 3.02 |
| **OB946_10945** | **taurine ABC transporter substrate-binding protein** | **3.00** |
| **OB946_14390** | hypothetical protein | 2.98 |
| **OB946_01700** | Holliday junction resolvase-like protein | 2.97 |
| **OB946_19680** |  | 2.92 |
| **OB946_09580** | MFS transporter | 2.90 |
| **OB946_07115** | hypothetical protein | 2.89 |
| **OB946_18880** | **sulfonate ABC transporter substrate-binding protein** | **2.89** |
| **OB946_17470** | TetR/AcrR family transcriptional regulator | 2.87 |
| **OB946_15225** | MFS transporter | 2.86 |
| **OB946_12365** | minor capsid protein | 2.86 |
| **OB946_18890** | **aliphatic sulfonate ABC transporter permease SsuC** | **2.84** |
| **OB946_08735** | MBL fold metallo-hydrolase | 2.82 |
| **OB946_03440** | lysophospholipid acyltransferase family protein | 2.80 |
| **OB946_07605** | magnesium-translocating P-type ATPase | 2.79 |
| **OB946_13200** | LysE/ArgO family amino acid transporter | 2.78 |
| **OB946_17840** | hypothetical protein | 2.73 |
| **OB946_10425** | metalloregulator ArsR/SmtB family transcription factor | 2.73 |
| **OB946_02780** | hypothetical protein | 2.72 |
| **OB946_12500** | benzoate 1%2C2-dioxygenase electron transfer component BenC | 2.72 |
| **OB946_12240** | hypothetical protein | 2.70 |
| **OB946_14925** | lipoprotein insertase outer membrane protein LolB | 2.69 |
| **OB946_10715** | SfnB family sulfur acquisition oxidoreductase | 2.69 |
| **OB946_14165** | hypothetical protein | 2.68 |
| **OB946_19640** |  | 2.66 |
| **OB946_11430** | acyl-CoA dehydrogenase family protein | 2.65 |
| **OB946_00325** | DUF523 domain-containing protein | 2.64 |
| **OB946_08060** | kynureninase | 2.64 |
| **OB946_07760** | type II 3-dehydroquinate dehydratase | 2.63 |
| **OB946_03935** | **multidrug efflux RND transporter permease subunit** | **2.63** |
| **OB946_10635** | PACE efflux transporter | 2.62 |
| **OB946_10265** | SRPBCC family protein | 2.61 |
| **OB946_10920** | NCS1 family nucleobase:cation symporter-1 | 2.59 |
| **OB946_14385** | RusA family crossover junction endodeoxyribonuclease | 2.59 |
| **OB946_19605** |  | 2.58 |
| **OB946_19305** |  | 2.53 |
| **OB946_12330** | hypothetical protein | 2.52 |
| **OB946_19340** |  | 2.52 |
| **OB946_17560** | PAS domain-containing sensor histidine kinase | 2.51 |
| **OB946_12390** | hypothetical protein | 2.51 |
| **OB946_16170** | **MacA family efflux pump subunit** | **2.50** |
| **OB946_14090** | PAAR domain-containing protein | 2.49 |
| **OB946_00625** | allantoicase | 2.49 |
| **OB946_19635** |  | 2.48 |
| **OB946_12835** | 3-(3-hydroxy-phenyl)propionate transporter MhpT | 2.48 |
| **OB946_05565** | hypothetical protein | 2.47 |
| **OB946_00330** | **4-hydroxyphenylpyruvate dioxygenase** | **2.47** |
| **OB946_16820** | fluoride efflux transporter CrcB | 2.46 |
| **OB946_03085** | DUF488 domain-containing protein | 2.46 |
| **OB946_06020** | DUF559 domain-containing protein | 2.46 |
| **OB946_06065** | putative metallopeptidase | 2.46 |
| **OB946_06145** | hypothetical protein | 2.45 |
| **OB946_18860** | **RcnB family protein** | **2.45** |
| **OB946_03505** | hypothetical protein | 2.44 |
| **OB946_18255** | hypothetical protein | 2.43 |
| **OB946_08930** | LysR family transcriptional regulator | 2.42 |
| **OB946_05200** | acinetobactin biosynthesis bifunctional isochorismatase/aryl carrier protein BasF | 2.42 |
| **OB946_12275** | phage minor head protein | 2.42 |
| **OB946_00985** | AzlC family ABC transporter permease | 2.41 |
| **OB946_14655** | bacteriohemerythrin | 2.41 |
| **OB946_10645** | integrase family protein | 2.41 |
| **OB946_08150** | hypothetical protein | 2.41 |
| **OB946_07860** | hypothetical protein | 2.41 |
| **OB946_19065** | hypothetical protein | 2.41 |
| **OB946_01120** | helix-turn-helix domain-containing protein | 2.40 |
| **OB946_09530** | CoA transferase subunit A | 2.40 |
| **OB946_04025** | SDR family oxidoreductase | 2.40 |
| **OB946_03965** | transglycosylase SLT domain-containing protein | 2.39 |
| **OB946_16315** | gluconokinase | 2.39 |
| **OB946_11495** | carboxymuconolactone decarboxylase family protein | 2.39 |
| **OB946_17730** |  | 2.38 |
| **OB946_02740** | DUF4184 family protein | 2.38 |
| **OB946_05080** | hypothetical protein | 2.38 |
| **OB946_01830** | TIGR03915 family putative DNA repair protein | 2.37 |
| **OB946_17675** | cation transporter | 2.37 |
| **OB946_19475** |  | 2.37 |
| **OB946_16335** | acetate kinase | 2.36 |
| **OB946_08065** | amino acid permease | 2.36 |
| **OB946_04865** | Lrp/AsnC family transcriptional regulator | 2.36 |
| **OB946_03370** | fatty acid desaturase family protein | 2.36 |
| **OB946_00040** | DUF6091 family protein | 2.36 |
| **OB946_12440** | hypothetical protein | 2.35 |
| **OB946_18240** | MFS transporter | 2.34 |
| **OB946_11915** | type VI secretion system contractile sheath small subunit | 2.33 |
| **OB946_19555** |  | 2.33 |
| **OB946_10965** | **monooxygenase** | **2.32** |
| **OB946_11375** | acyl-CoA dehydrogenase family protein | 2.31 |
| **OB946_15410** | hypothetical protein | 2.30 |
| **OB946_16320** | gluconate:H+ symporter | 2.30 |
| **OB946_04410** | sulfate ABC transporter permease subunit CysW | 2.29 |
| **OB946_17930** | phage baseplate assembly protein V | 2.26 |
| **OB946_11230** | iron-containing redox enzyme family protein | 2.26 |
| **OB946_09715** | lipoyl synthase | 2.25 |
| **OB946_00825** | putative porin | 2.25 |
| **OB946_01125** | GNAT family N-acetyltransferase | 2.24 |
| **OB946_00910** | cation acetate symporter | 2.24 |
| **OB946_09705** | alpha-ketoacid dehydrogenase subunit beta | 2.24 |
| **OB946_11475** | 4-hydroxybenzoate 3-monooxygenase | 2.24 |
| **OB946_16800** | SfnB family sulfur acquisition oxidoreductase | 2.23 |
| **OB946_08145** | hypothetical protein | 2.21 |
| **OB946_15505** | FAD-dependent tricarballylate dehydrogenase TcuA | 2.21 |
| **OB946_01630** | hemerythrin domain-containing protein | 2.21 |
| **OB946_19390** |  | 2.21 |
| **OB946_16175** | MacB family efflux pump subunit | 2.20 |
| **OB946_09150** | FAD-binding oxidoreductase | 2.20 |
| **OB946_09615** | fumarate reductase/succinate dehydrogenase flavoprotein subunit | 2.20 |
| **OB946_12355** | helix-turn-helix domain-containing protein | 2.20 |
| **OB946_13895** | LysR family transcriptional regulator | 2.19 |
| **OB946_08050** | GNAT family N-acetyltransferase | 2.19 |
| **OB946_09830** | hypothetical protein | 2.19 |
| **OB946_05365** | hypothetical protein | 2.19 |
| **OB946_01255** | acetyl-CoA hydrolase/transferase family protein | 2.18 |
| **OB946_12105** | Lrp/AsnC family transcriptional regulator | 2.18 |
| **OB946_13915** | hypothetical protein | 2.17 |
| **OB946_11500** | NAD-dependent succinate-semialdehyde dehydrogenase | 2.17 |
| **OB946_11005** | malonate transporter subunit MadL | 2.16 |
| **OB946_19500** |  | 2.16 |
| **OB946_06790** | paraquat-inducible protein A | 2.15 |
| **OB946_13430** | sulfate adenylyltransferase subunit CysD | 2.15 |
| **OB946_10640** | LysR family transcriptional regulator | 2.14 |
| **OB946_04840** | TetR/AcrR family transcriptional regulator | 2.14 |
| **OB946_03170** | DUF1853 family protein | 2.14 |
| **OB946_00940** | TetR/AcrR family transcriptional regulator | 2.13 |
| **OB946_00320** | MATE family efflux transporter | 2.13 |
| **OB946_01705** | hypothetical protein | 2.13 |
| **OB946_15470** | AraC family transcriptional regulator | 2.12 |
| **OB946_01615** | lipase secretion chaperone | 2.12 |
| **OB946_14805** | hypothetical protein | 2.12 |
| **OB946_11020** | biotin-independent malonate decarboxylase subunit gamma | 2.12 |
| **OB946_08590** | flavin reductase family protein | 2.12 |
| **OB946_18995** | LysR family transcriptional regulator | 2.11 |
| **OB946_00645** | hydroxyisourate hydrolase | 2.11 |
| **OB946_09500** | MFS transporter | 2.11 |
| **OB946_19655** |  | 2.11 |
| **OB946_08015** | regulatory protein RecX | 2.11 |
| **OB946_11530** | flavin reductase family protein | 2.11 |
| **OB946_04655** | hypothetical protein | 2.11 |
| **OB946_02270** | DUF3108 domain-containing protein | 2.10 |
| **OB946_11775** | dihydrodipicolinate synthase family protein | 2.10 |
| **OB946_09455** | anthranilate 1%2C2-dioxygenase large subunit | 2.10 |
| **OB946_15230** | HlyD family secretion protein | 2.10 |
| **OB946_08085** | SulP family inorganic anion transporter | 2.10 |
| **OB946_12360** | MFS transporter | 2.10 |
| **OB946_04420** | alpha/beta hydrolase | 2.09 |
| **OB946_08470** | right-handed parallel beta-helix repeat-containing protein | 2.09 |
| **OB946_19515** |  | 2.08 |
| **OB946_12735** | NAD(P)/FAD-dependent oxidoreductase | 2.08 |
| **OB946_07435** | hypothetical protein | 2.08 |
| **OB946_07820** | MoaD/ThiS family protein | 2.08 |
| **OB946_18125** | esterase-like activity of phytase family protein | 2.08 |
| **OB946_00185** | hypothetical protein | 2.07 |
| **OB946_12810** | MFS transporter | 2.07 |
| **OB946_01740** | arginine N-succinyltransferase | 2.06 |
| **OB946_02925** | hypothetical protein | 2.06 |
| **OB946_17700** |  | 2.05 |
| **OB946_11885** | hypothetical protein | 2.05 |
| **OB946_04580** | hypothetical protein | 2.05 |
| **OB946_19645** |  | 2.05 |
| **OB946_13800** | BCCT family transporter | 2.05 |
| **OB946_16295** | class I SAM-dependent methyltransferase | 2.05 |
| **OB946_17295** | YMGG-like glycine zipper-containing protein | 2.05 |
| **OB946_13650** | MFS transporter | 2.05 |
| **OB946_10775** | L-valine transporter subunit YgaH | 2.04 |
| **OB946_07130** | type 1 glutamine amidotransferase domain-containing protein | 2.04 |
| **OB946_08045** | hypothetical protein | 2.04 |
| **OB946_04585** | hypothetical protein | 2.04 |
| **OB946_00430** | ATP-binding protein | 2.04 |
| **OB946_15235** | pyridoxal-phosphate dependent enzyme | 2.04 |
| **OB946_09315** | fimbrial protein | 2.03 |
| **OB946_09540** | TIGR00366 family protein | 2.03 |
| **OB946_14855** | LysR family transcriptional regulator | 2.03 |
| **OB946_11155** | amino acid ABC transporter permease | 2.02 |
| **OB946_08540** | MFS transporter | 2.02 |
| **OB946_16330** | phosphogluconate dehydratase | 2.02 |
| **OB946_06305** | multidrug efflux transcriptional repressor AdeL | 2.02 |
| **OB946_11345** | **PaaI family thioesterase** | **2.01** |
| **OB946_11385** | SDR family oxidoreductase | 2.01 |
| **OB946_04240** | rhombotarget A | 2.01 |
| **OB946_02785** | sensor histidine kinase efflux regulator BaeS | 2.01 |
| **OB946_07565** | OmpW family outer membrane protein | 2.01 |
| **OB946_02970** | SMR family transporter | 2.01 |
| **OB946_06850** |  | 2.00 |
| **OB946_13195** | LysR family transcriptional regulator ArgP | 2.00 |
| **OB946_12955** | Lrp/AsnC family transcriptional regulator | 2.00 |
| **OB946_13735** | nicotinamide riboside transporter PnuC | 2.00 |
| **OB946_14245** | hypothetical protein | -2.00 |
| **OB946_08240** | cytochrome d ubiquinol oxidase subunit II | -2.01 |
| **OB946_08245** | cytochrome ubiquinol oxidase subunit I | -2.01 |
| **OB946_08630** | 3-oxoacid CoA-transferase subunit B | -2.01 |
| **OB946_03775** | universal stress protein | -2.02 |
| **OB946_19130** | 50S ribosomal protein L34 | -2.02 |
| **OB946_02005** | 30S ribosomal protein S10 | -2.02 |
| **OB946_11905** | type VI secretion system tube protein Hcp | -2.03 |
| **OB946_11485** | NAD(P)H-dependent oxidoreductase | -2.03 |
| **OB946_16105** | hypothetical protein | -2.04 |
| **OB946_12560** | hypothetical protein | -2.04 |
| **OB946_19040** | DMT family protein | -2.04 |
| **OB946_03790** | carbamoyl-phosphate synthase large subunit | -2.04 |
| **OB946_15320** | NADH-quinone oxidoreductase subunit NuoH | -2.05 |
| **OB946_06180** | hypothetical protein | -2.05 |
| **OB946_08235** | cytochrome bd-I oxidase subunit CydX | -2.05 |
| **OB946_11955** | biotin carboxylase N-terminal domain-containing protein | -2.06 |
| **OB946_09945** | hypothetical protein | -2.06 |
| **OB946_11195** | LysE family translocator | -2.06 |
| **OB946_03095** | YegP family protein | -2.08 |
| **OB946_06320** | ABC transporter permease | -2.09 |
| **OB946_05530** | hypothetical protein | -2.09 |
| **OB946_06925** | hypothetical protein | -2.10 |
| **OB946_15330** | NADH-quinone oxidoreductase subunit NuoF | -2.10 |
| **OB946_03020** | translational GTPase TypA | -2.10 |
| **OB946_17110** | type II secretion system F family protein | -2.10 |
| **OB946_15300** | NADH-quinone oxidoreductase subunit L | -2.10 |
| **OB946_15290** | NADH-quinone oxidoreductase subunit NuoN | -2.10 |
| **OB946_05505** | phage terminase large subunit | -2.12 |
| **OB946_04395** | ornithine uptake porin CarO type 1 | -2.12 |
| **OB946_05560** | hypothetical protein | -2.13 |
| **OB946_15325** | NADH-quinone oxidoreductase subunit NuoG | -2.13 |
| **OB946_01580** | **type IV pilin protein** | **-2.14** |
| **OB946_04995** | EAL domain-containing protein | -2.14 |
| **OB946_09270** | virulence factor TspB C-terminal domain-related protein | -2.15 |
| **OB946_01455** | **type IV pilus secretin PilQ family protein** | **-2.15** |
| **OB946_01850** | acyl-CoA dehydrogenase C-terminal domain-containing protein | -2.16 |
| **OB946_09225** | hypothetical protein | -2.17 |
| **OB946_05525** | Gp49 family protein | -2.17 |
| **OB946_05330** | hypothetical protein | -2.18 |
| **OB946_07930** | nitrogen regulation protein NR(I) | -2.18 |
| **OB946_01575** | VWA domain-containing protein | -2.19 |
| **OB946_10865** | chromate transporter | -2.19 |
| **OB946_18220** | DNA-processing protein DprA | -2.20 |
| **OB946_15850** | hypothetical protein | -2.20 |
| **OB946_01565** | **PilW family protein** | **-2.20** |
| **OB946_02000** | type 1 glutamine amidotransferase | -2.20 |
| **OB946_01555** | **GspH/FimT family pseudopilin** | **-2.21** |
| **OB946_09210** | hypothetical protein | -2.27 |
| **OB946_12535** | alkyl hydroperoxide reductase subunit C | -2.27 |
| **OB946_06675** | glycosyltransferase | -2.29 |
| **OB946_11225** | catalase HPII | -2.30 |
| **OB946_03135** | methyl-accepting chemotaxis protein | -2.30 |
| **OB946_07655** | MaoC/PaaZ C-terminal domain-containing protein | -2.32 |
| **OB946_02010** | 50S ribosomal protein L3 | -2.32 |
| **OB946_09255** | hypothetical protein | -2.34 |
| **OB946_09285** | hypothetical protein | -2.35 |
| **OB946_08650** | muconate/chloromuconate family cycloisomerase | -2.36 |
| **OB946_13875** | Slam-dependent surface lipoprotein | -2.36 |
| **OB946_02020** | 50S ribosomal protein L23 | -2.37 |
| **OB946_07615** | benzoate/H(+) symporter BenE family transporter | -2.38 |
| **OB946_16150** | DUF4124 domain-containing protein | -2.40 |
| **OB946_13560** | hypothetical protein | -2.41 |
| **OB946_02015** | 50S ribosomal protein L4 | -2.42 |
| **OB946_09120** | multidrug efflux RND transporter periplasmic adaptor subunit AdeA | -2.43 |
| **OB946_15250** | mechanosensitive ion channel | -2.43 |
| **OB946_07650** | 3-oxoacyl-ACP reductase | -2.48 |
| **OB946_01450** | **pilus assembly protein PilP** | **-2.50** |
| **OB946_11650** | **PaaI family thioesterase** | **-2.50** |
| **OB946_14625** | **type IV pilus twitching motility protein PilT** | **-2.51** |
| **OB946_08685** | aldehyde dehydrogenase (NADP(+)) | -2.53 |
| **OB946_04335** | multidrug effflux MFS transporter | -2.53 |
| **OB946_01050** | MFS transporter | -2.54 |
| **OB946_11660** | **phenylacetic acid degradation operon negative regulatory protein PaaX** | **-2.54** |
| **OB946_11685** | enoyl-CoA hydratase-related protein | -2.54 |
| **OB946_15715** | ribosome-associated translation inhibitor RaiA | -2.55 |
| **OB946_10780** | tautomerase family protein | -2.56 |
| **OB946_05545** | hypothetical protein | -2.58 |
| **OB946_08230** | cyd operon YbgE family protein | -2.59 |
| **OB946_03145** | hypothetical protein | -2.63 |
| **OB946_08695** | alpha/beta hydrolase | -2.65 |
| **OB946_05540** | Ish1 domain-containing protein | -2.65 |
| **OB946_09165** | hypothetical protein | -2.73 |
| **OB946_11680** | **2-(1%2C2-epoxy-1%2C2-dihydrophenyl)acetyl-CoA isomerase PaaG** | **-2.75** |
| **OB946_05255** | DUF3015 family protein | -2.81 |
| **OB946_09260** | zonular occludens toxin domain-containing protein | -2.88 |
| **OB946_10590** | fimbrial protein | -2.89 |
| **OB946_04785** | type I-F CRISPR-associated endoribonuclease Cas6/Csy4 | -2.93 |
| **OB946_00365** | zinc metallochaperone GTPase ZigA | -2.94 |
| **OB946_04780** | type I-F CRISPR-associated protein Csy3 | -2.95 |
| **OB946_09660** | FAD/NAD(P)-binding oxidoreductase | -3.04 |
| **OB946_03140** | Hpt domain-containing protein | -3.10 |
| **OB946_09670** | MBL fold metallo-hydrolase | -3.17 |
| **OB946_05465** | hypothetical protein | -3.23 |
| **OB946_08120** | PEGA domain-containing protein | -3.26 |
| **OB946_09425** | isochorismatase family protein | -3.29 |
| **OB946_11460** | ATP-binding cassette domain-containing protein | -3.32 |
| **OB946_01570** | hypothetical protein | -3.32 |
| **OB946_14630** | **PilT/PilU family type 4a pilus ATPase** | **-3.37** |
| **OB946_08640** | catechol 1%2C2-dioxygenase | -3.46 |
| **OB946_11655** | DapH/DapD/GlmU-related protein | -3.52 |
| **OB946_07645** | acetyl-CoA C-acetyltransferase | -3.52 |
| **OB946_00200** | MFS transporter | -3.54 |
| **OB946_15000** | peptidoglycan-binding protein LysM | -3.65 |
| **OB946_08645** | muconolactone Delta-isomerase | -3.74 |
| **OB946_11665** | **phenylacetate--CoA ligase PaaK** | **-3.76** |
| **OB946_09280** | hypothetical protein | -4.01 |
| **OB946_08785** | hydrolase | -4.32 |
| **OB946_09205** | hypothetical protein | -4.46 |
| **OB946_10625** | hypothetical protein | -4.57 |
| **OB946_08780** | putative quinol monooxygenase | -4.72 |
| **OB946_11675** | 3-hydroxyacyl-CoA dehydrogenase | -4.73 |
| **OB946_09665** |  | -5.13 |
| **OB946_07585** | universal stress protein | -5.16 |
| **OB946_11670** | 3-oxoadipyl-CoA thiolase | -6.25 |
| **OB946_10620** | DMT family transporter | -7.32 |
| **OB946_01505** | **pilin** | **-9.07** |
| **OB946_09200** | major capsid protein | -14.46 |
| **OB946_09275** | major capsid protein | -14.52 |

a| Fold change cutoff: 2-fold *P* value < 0.01. Differential expression was calculated with DESeq2.
