# Supplementary material for: Diclofenac sensitizes multi-drug resistant Acinetobacter baumannii to colistin
Source: PLoS Pathog. 2024 Nov 21;20(11):e1012705. doi: 10.1371/journal.ppat.1012705 (PMC11620633; doi:10.1371/journal.ppat.1012705)
Supplement: S8 Table — (DOCX) [file ppat.1012705.s018.docx]

**Table S8: Differentially expressed genes in ARC6851 in colistin + diclofenac treatment vs diclofenac.**

| **Accession** | **Annotated gene** | **Fold change^a^** |
| --- | --- | --- |
| **OB946_12790** | **SDR family oxidoreductase** | **11.54** |
| **OB946_12785** | **NAD(P)/FAD-dependent oxidoreductase** | **10.04** |
| **OB946_12795** | alpha/beta hydrolase | 5.22 |
| **OB946_00885** | FMN reductase | 4.67 |
| **OB946_09560** | hypothetical protein | 4.64 |
| **OB946_09895** | sulfite exporter TauE/SafE family protein | 4.08 |
| **OB946_01760** | hypothetical protein | 3.97 |
| **OB946_18875** | sulfonate ABC transporter substrate-binding protein | 3.86 |
| **OB946_15220** | TetR/AcrR family transcriptional regulator | 3.72 |
| **OB946_18890** | **aliphatic sulfonate ABC transporter permease SsuC** | **3.35** |
| **OB946_00880** | **dimethyl sulfone monooxygenase SfnG** | **3.23** |
| **OB946_18865** | **RcnB family protein** | **3.07** |
| **OB946_04880** | substrate-binding domain-containing protein | 2.92 |
| **OB946_12420** | transposase | 2.88 |
| **OB946_15495** | substrate-binding domain-containing protein | 2.82 |
| **OB946_10965** | **monooxygenase** | **2.77** |
| **OB946_15450** | DUF1852 domain-containing protein | 2.77 |
| **OB946_07300** | hypothetical protein | 2.66 |
| **OB946_15490** | MFS transporter | 2.65 |
| **OB946_17300** | hypothetical protein | 2.63 |
| **OB946_16045** | RidA family protein | 2.58 |
| **OB946_04425** | sulfate ABC transporter substrate-binding protein | 2.56 |
| **OB946_04895** | phosphate ABC transporter ATP-binding protein PstB | 2.54 |
| **OB946_13200** | LysE/ArgO family amino acid transporter | 2.52 |
| **OB946_02760** | **RcnB family protein** | **2.52** |
| **OB946_12390** | hypothetical protein | 2.46 |
| **OB946_18880** | **sulfonate ABC transporter substrate-binding protein** | **2.46** |
| **OB946_09460** | anthranilate 1%2C2-dioxygenase small subunit | 2.44 |
| **OB946_09715** | lipoyl synthase | 2.37 |
| **OB946_03085** | DUF488 domain-containing protein | 2.37 |
| **OB946_02780** | hypothetical protein | 2.37 |
| **OB946_13205** | cation diffusion facilitator family transporter | 2.36 |
| **OB946_09940** | GNAT family N-acetyltransferase | 2.36 |
| **OB946_07435** | hypothetical protein | 2.35 |
| **OB946_18255** | hypothetical protein | 2.35 |
| **OB946_12440** | hypothetical protein | 2.32 |
| **OB946_18800** | hypothetical protein | 2.32 |
| **OB946_09500** | MFS transporter | 2.29 |
| **OB946_17865** | site-specific integrase | 2.28 |
| **OB946_02885** | **peroxiredoxin** | **2.27** |
| **OB946_06760** | hypothetical protein | 2.26 |
| **OB946_03965** | transglycosylase SLT domain-containing protein | 2.21 |
| **OB946_10945** | **taurine ABC transporter substrate-binding protein** | **2.19** |
| **OB946_13800** | BCCT family transporter | 2.18 |
| **OB946_18985** | hypothetical protein | 2.17 |
| **OB946_04890** | phosphate ABC transporter permease PstA | 2.16 |
| **OB946_03935** | **multidrug efflux RND transporter permease subunit** | **2.15** |
| **OB946_08435** | 3-carboxy-cis%2Ccis-muconate cycloisomerase | 2.12 |
| **OB946_10395** | hypothetical protein | 2.11 |
| **OB946_15410** | hypothetical protein | 2.11 |
| **OB946_06850** |  | 2.10 |
| **OB946_13430** | sulfate adenylyltransferase subunit CysD | 2.10 |
| **OB946_09455** | anthranilate 1%2C2-dioxygenase large subunit | 2.10 |
| **OB946_07115** | hypothetical protein | 2.09 |
| **OB946_18885** | **FMNH2-dependent alkanesulfonate monooxygenase** | **2.09** |
| **OB946_04885** | phosphate ABC transporter permease subunit PstC | 2.09 |
| **OB946_00595** | lysozyme inhibitor LprI family protein | 2.08 |
| **OB946_12445** | LysE family transporter | 2.07 |
| **OB946_17670** | signal peptidase II | 2.07 |
| **OB946_18860** | **RcnB family protein** | **2.05** |
| **OB946_02740** | DUF4184 family protein | 2.05 |
| **OB946_18895** | ATP-binding cassette domain-containing protein | 2.05 |
| **OB946_00240** | hypothetical protein | 2.04 |
| **OB946_18980** | hypothetical protein | 2.04 |
| **OB946_09465** | anthranilate 1%2C2-dioxygenase electron transfer component AntC | 2.04 |
| **OB946_11020** | biotin-independent malonate decarboxylase subunit gamma | 2.04 |
| **OB946_13210** | MerR family transcriptional regulator | 2.02 |
| **OB946_12205** | site-specific integrase | 2.01 |
| **OB946_02160** | membrane protein | 2.01 |
| **OB946_12830** | MarR family transcriptional regulator | -2.01 |
| **OB946_19440** |  | -2.02 |
| **OB946_10780** | tautomerase family protein | -2.03 |
| **OB946_19240** |  | -2.03 |
| **OB946_01575** | VWA domain-containing protein | -2.03 |
| **OB946_15360** | hypothetical protein | -2.04 |
| **OB946_11975** | divalent metal cation transporter | -2.04 |
| **OB946_04870** | thiamine pyrophosphate-binding protein | -2.04 |
| **OB946_13975** | hypothetical protein | -2.05 |
| **OB946_18025** | TorF family putative porin | -2.06 |
| **OB946_08960** | phosphoribosyltransferase family protein | -2.07 |
| **OB946_06835** | acyl-CoA desaturase | -2.09 |
| **OB946_05255** | DUF3015 family protein | -2.09 |
| **OB946_11660** | phenylacetic acid degradation operon negative regulatory protein PaaX | -2.10 |
| **OB946_08205** | hypothetical protein | -2.12 |
| **OB946_11390** | 3-hydroxyacyl-CoA dehydrogenase | -2.13 |
| **OB946_03135** | methyl-accepting chemotaxis protein | -2.13 |
| **OB946_00385** | urocanate hydratase | -2.13 |
| **OB946_04045** | formate dehydrogenase accessory sulfurtransferase FdhD | -2.14 |
| **OB946_15250** | mechanosensitive ion channel | -2.16 |
| **OB946_03495** | hypothetical protein | -2.16 |
| **OB946_01450** | **pilus assembly protein PilP** | **-2.16** |
| **OB946_08035** | YcgJ family protein | -2.17 |
| **OB946_07615** | benzoate/H(+) symporter BenE family transporter | -2.17 |
| **OB946_17940** | baseplate J/gp47 family protein | -2.17 |
| **OB946_07685** | thioesterase family protein | -2.19 |
| **OB946_14725** | threonine export protein RhtC | -2.19 |
| **OB946_05105** | TetR/AcrR family transcriptional regulator | -2.20 |
| **OB946_03145** | hypothetical protein | -2.20 |
| **OB946_07570** | hypothetical protein | -2.21 |
| **OB946_04995** | EAL domain-containing protein | -2.22 |
| **OB946_09285** | hypothetical protein | -2.24 |
| **OB946_09255** | hypothetical protein | -2.25 |
| **OB946_01455** | **type IV pilus secretin PilQ family protein** | **-2.30** |
| **OB946_04335** | multidrug effflux MFS transporter | -2.30 |
| **OB946_09310** | hypothetical protein | -2.31 |
| **OB946_06025** | hypothetical protein | -2.31 |
| **OB946_10590** | fimbrial protein | -2.33 |
| **OB946_09425** | isochorismatase family protein | -2.35 |
| **OB946_00365** | zinc metallochaperone GTPase ZigA | -2.36 |
| **OB946_09225** | hypothetical protein | -2.36 |
| **OB946_08785** | hydrolase | -2.37 |
| **OB946_08690** | hypothetical protein | -2.38 |
| **OB946_08495** | DUF6691 family protein | -2.40 |
| **OB946_16105** | hypothetical protein | -2.41 |
| **OB946_19130** | 50S ribosomal protein L34 | -2.41 |
| **OB946_01565** | **PilW family protein** | **-2.43** |
| **OB946_11685** | enoyl-CoA hydratase-related protein | -2.46 |
| **OB946_12075** | MFS transporter | -2.48 |
| **OB946_14625** | **type IV pilus twitching motility protein PilT** | -2.48 |
| **OB946_03685** |  | -2.50 |
| **OB946_01570** | hypothetical protein | -2.52 |
| **OB946_11680** | 2-(1%2C2-epoxy-1%2C2-dihydrophenyl)acetyl-CoA isomerase PaaG | -2.54 |
| **OB946_10625** | hypothetical protein | -2.56 |
| **OB946_11650** | PaaI family thioesterase | -2.56 |
| **OB946_05345** |  | -2.56 |
| **OB946_08365** | nuclear transport factor 2 family protein | -2.59 |
| **OB946_16150** | DUF4124 domain-containing protein | -2.65 |
| **OB946_04785** | type I-F CRISPR-associated endoribonuclease Cas6/Csy4 | -2.67 |
| **OB946_10865** | chromate transporter | -2.67 |
| **OB946_07645** | acetyl-CoA C-acetyltransferase | -2.67 |
| **OB946_01560** | **type IV pilus modification protein PilV** | **-2.70** |
| **OB946_17855** | hypothetical protein | -2.71 |
| **OB946_07585** | universal stress protein | -2.72 |
| **OB946_04780** | type I-F CRISPR-associated protein Csy3 | -2.81 |
| **OB946_15000** | peptidoglycan-binding protein LysM | -2.83 |
| **OB946_03140** | Hpt domain-containing protein | -2.84 |
| **OB946_11665** | phenylacetate--CoA ligase PaaK | -2.85 |
| **OB946_19385** |  | -2.94 |
| **OB946_10620** | DMT family transporter | -3.12 |
| **OB946_05540** | Ish1 domain-containing protein | -3.17 |
| **OB946_09280** | hypothetical protein | -3.21 |
| **OB946_14630** | **PilT/PilU family type 4a pilus ATPase** | **-3.28** |
| **OB946_06180** | hypothetical protein | -3.28 |
| **OB946_01555** | **GspH/FimT family pseudopilin** | **-3.30** |
| **OB946_11655** | DapH/DapD/GlmU-related protein | -3.40 |
| **OB946_09665** |  | -3.41 |
| **OB946_11675** | 3-hydroxyacyl-CoA dehydrogenase | -3.42 |
| **OB946_00200** | MFS transporter | -3.43 |
| **OB946_09205** | hypothetical protein | -4.19 |
| **OB946_11670** | 3-oxoadipyl-CoA thiolase | -4.72 |
| **OB946_01505** | **pilin** | **-5.91** |
| **OB946_09275** | major capsid protein | -9.25 |
| **OB946_09200** | major capsid protein | -9.35 |

a| Fold change cutoff: 2-fold *P* value < 0.01. Differential expression was calculated with DESeq2.
