# Supplementary material for: Diclofenac sensitizes multi-drug resistant Acinetobacter baumannii to colistin
Source: PLoS Pathog. 2024 Nov 21;20(11):e1012705. doi: 10.1371/journal.ppat.1012705 (PMC11620633; doi:10.1371/journal.ppat.1012705)
Supplement: S9 Table — (DOCX) [file ppat.1012705.s019.docx]

**Table S9: Differentially expressed proteins in ARC6851 in colistin + diclofenac treatment vs DMSO.**

| **Accession** | **Annotated protein** | **Fold change^a^** |
| --- | --- | --- |
| UYC77247.1 | hypothetical protein | 9.09 |
| UYC76564.1 | Transcriptional regulator, AcrR family | 8.53 |
| UYC78812.1 | Beta-ketoadipate enol-lactone hydrolase (EC 3.1.1.24) | 5.38 |
| **UYC76578.1** | **NADH-ubiquinone oxidoreductase chain M (EC 1.6.5.3)** | **4.92** |
| **UYC75585.1** | **Putative sulfate permease** | **4.62** |
| UYC77596.1 | hypothetical protein | 4.34 |
| UYC76922.1 | CDP-diacylglycerol--glycerol-3-phosphate 3-phosphatidyltransferase (EC 2.7.8.5) | 3.67 |
| UYC75975.1 | Uncharacterized metal ion transporter YcsG, Mn(2+)/Fe(2+) NRAMP family | 3.59 |
| UYC78867.1 | TetR/AcrR family transcriptional regulator | 3.56 |
| UYC78269.1;UYC78378.1 | minor capsid protein | 3.53 |
| UYC78817.1 | 3-dehydroshikimate dehydratase (EC 4.2.1.118) | 3.17 |
| UYC75736.1 | Glutamate/aspartate ABC transporter, permease protein GltK (TC 3.A.1.3.4) | 3.14 |
| **UYC76566.1** | **Membrane fusion component of MSF-type tripartite multidrug efflux system** | **3.00** |
| UYC78767.1 | hypothetical protein | 2.64 |
| UYC77309.1 | DNA-3-methyladenine glycosylase I | 2.63 |
| UYC77134.1 | DedA protein | 2.58 |
| UYC76190.1 | Arginine exporter protein ArgO | 2.48 |
| UYC77766.1 | Putative transmembrane protein | 2.46 |
| UYC76238.1 | 2-oxoglutarate dehydrogenase complex, dehydrogenase component | 2.43 |
| UYC78596.1 | FIG00352022: hypothetical protein | 2.37 |
| UYC76037.1 | Transcriptional regulator, HxlR family | 2.33 |
| UYC75575.1 | Bis(5'-nucleosyl)-tetraphosphatase (asymmetrical) (EC 3.6.1.17) | 2.32 |
| UYC75915.1 | ATP-dependent helicase | 2.32 |
| UYC76930.1 | hypothetical protein | 2.22 |
| **UYC76504.1** | **Multimeric flavodoxin WrbA** | **2.16** |
| UYC76492.1 | FIG00350262: hypothetical protein | 2.14 |
| **UYC78418.1** | **Probable transcription regulator protein of MDR efflux pump cluster** | **2.08** |
| UYC76035.1 | hypothetical protein | 2.00 |
| **UYC75917.1** | **Acyl-coenzyme A thioesterase PaaD (Pse.pu.) (E. coli PaaI)** | **2.00** |
| UYC78003.1 | D-serine/D-alanine/glycine transporter | -2.00 |
| UYC76193.1 | FIG00350187: hypothetical protein | -2.01 |
| UYC78169.1 | 5'-nucleotidase SurE (EC 3.1.3.5) | -2.02 |
| UYC79102.1 | Histone acetyltransferase HPA2 and related acetyltransferases | -2.04 |
| UYC77125.1 | Cell division protein | -2.04 |
| UYC76152.1 | Oxidoreductase | -2.04 |
| UYC78598.1 | Acetyltransferase, GNAT family | -2.06 |
| UYC78159.1 | Large repetitive protein | -2.12 |
| UYC77384.1 | Nucleoside-binding outer membrane protein | -2.16 |
| UYC76795.1 | LSU ribosomal protein L33p @ LSU ribosomal protein L33p, zinc-independent | -2.16 |
| UYC75868.1 | 3-hydroxyacyl-CoA dehydrogenase | -2.18 |
| UYC77509.1 | hypothetical protein OB946_01300 | -2.20 |
| UYC77005.1 | hypothetical protein | -2.22 |
| UYC76852.1 | Type III effector HopPmaJ | -2.24 |
| UYC76907.1 | Leader peptidase (Prepilin peptidase) (EC 3.4.23.43) / N-methyltransferase (EC 2.1.1.-) | -2.25 |
| UYC76219.1 | Urease accessory protein UreE | -2.25 |
| UYC77374.1 | hypothetical protein OB946_00585 | -2.26 |
| **UYC76455.1** | **Type IV pilus assembly ATPase component PilU** | **-2.27** |
| **UYC77535.1** | **Type IV pilus biogenesis protein PilQ** | **-2.31** |
| UYC76020.1 | hypothetical protein OB946_12265 | -2.33 |
| UYC79027.1 | hypothetical protein OB946_02540 | -2.34 |
| UYC77207.1 | 1,6-anhydro-N-acetylmuramyl-L-alanine amidase | -2.38 |
| UYC78379.1 | hypothetical protein OB946_06090 | -2.44 |
| UYC75698.1 | hypothetical protein | -2.53 |
| UYC76183.1 | FIG00350110: hypothetical protein | -2.55 |
| UYC76926.1 | hypothetical protein OB946_06090 | -2.57 |
| **UYC77836.1** | **Twitching motility protein PilH** | **-2.57** |
| UYC79047.1 | hypothetical protein OB946_17215 | -2.58 |
| **UYC79020.1** | **Type IV pilus biogenesis protein PilP** | **-2.60** |
| UYC76816.1 | hypothetical protein | -2.61 |
| UYC78114.1 | FKBP-type peptidyl-prolyl cis-trans isomerase SlyD (EC 5.2.1.8) | -2.63 |
| **UYC77534.1** | **Type IV pilus biogenesis protein PilO** | **-2.69** |
| UYC76426.1 | YfdQ family protein | -2.71 |
| UYC76450.1 | OsmC/Ohr family protein | -2.75 |
| **UYC77533.1** | **Type IV pilus biogenesis protein PilN** | **-2.84** |
| UYC76525.1 | LSU ribosomal protein L32p @ LSU ribosomal protein L32p, zinc-independent | -2.91 |
| **UYC77532.1** | **Type IV pilus biogenesis protein PilM** | **-2.98** |
| UYC78428.1 | Type II secretory pathway, ATPase PulE/Tfp pilus assembly pathway, ATPase PilB | -3.00 |
| UYC75926.1 | 1,2-phenylacetyl-CoA epoxidase, subunit D (EC 1.14.13.149) | -3.02 |
| UYC77644.1 | SSU ribosomal protein S19p (S15e) | -3.07 |
| **UYC77835.1** | **Twitching motility protein PilG** | **-3.11** |
| UYC76608.1 | hypothetical protein | -3.14 |
| UYC76488.1 | Protoporphyrinogen IX oxidase, novel form, HemJ (EC 1.3.-.-) | -3.20 |
| UYC76441.1 | endonuclease/exonuclease/phosphatase family protein | -3.24 |
| UYC77519.1 | Uncharacterized amino acid permease, GabP family | -3.27 |
| UYC78424.1 | Uncharacterized protease YegQ | -3.31 |
| **UYC76906.1** | **Type IV fimbrial assembly protein PilC** | **-3.68** |
| UYC77157.1 | D-serine/D-alanine/glycine transporter | -3.79 |
| UYC75670.1 | MotA/TolQ/ExbB proton channel family protein | -3.83 |
| UYC75523.1 | Aspartate ammonia-lyase (EC 4.3.1.1) | -4.20 |
| UYC78695.1 | GTP 3',8-cyclase (EC 4.1.99.22) | -4.66 |
| UYC79070.1 | PEGA domain-containing protein | -4.74 |
| UYC77025.1 | BRCT domain-containing protein | -5.19 |
| UYC78168.1 | hypothetical protein | -5.22 |
| UYC78954.1;UYC78945.1 | hypothetical protein OB946_09210 | -5.46 |
| UYC77840.1 | hypothetical protein | -6.10 |
| **UYC77837.1** | **Type IV pili signal transduction protein PilI** | **-6.70** |
| **UYC77838.1** | **Type IV pilus biogenesis protein PilJ** | -6.86 |
| UYC78963.1;UYC78949.1 | zonular occludens toxin domain-containing protein | -7.16 |
| **UYC77558.1** | **Type IV fimbrial biogenesis protein PilY1** | **-7.90** |
| UYC76460.1 | Hemerythrin domain protein | -10.26 |
| **UYC77545.1** | **Type IV pilin PilA** | **-10.95** |
| **UYC77839.1** | **Twitching motility protein PilG** | **-18.79** |
| UYC76989.1 | Helix-turn-helix, Fis-type | -22.74 |

**a|** Fold change cutoff: 2-fold with a p-value < 0.05. Student’s unpaired *t* test.
