# Supplementary material for: Diclofenac sensitizes multi-drug resistant Acinetobacter baumannii to colistin
Source: PLoS Pathog. 2024 Nov 21;20(11):e1012705. doi: 10.1371/journal.ppat.1012705 (PMC11620633; doi:10.1371/journal.ppat.1012705)
Supplement: S10 Table — (DOCX) [file ppat.1012705.s020.docx]

**Table S10: Differentially expressed proteins in ARC6851 in colistin + diclofenac treatment vs colistin.**

| **Accession** | **Annotated protein** | **Fold change^a^** |
| --- | --- | --- |
| UYC76564.1 | Transcriptional regulator, AcrR family | 8.84 |
| UYC79064.1 | Glycine-rich cell wall structural protein precursor | 5.42 |
| UYC76804.1 | hypothetical protein | 4.88 |
| UYC77596.1 | hypothetical protein | 4.18 |
| UYC78867.1 | TetR/AcrR family transcriptional regulator | 3.27 |
| **UYC76566.1** | **Membrane fusion component of MSF-type tripartite multidrug efflux system** | **2.88** |
| UYC76190.1 | Arginine exporter protein ArgO | 2.85 |
| UYC75585.1 | Putative sulfate permease | 2.75 |
| UYC79067.1 | Mg(2+) transport ATPase, P-type (EC 3.6.3.2) | 2.67 |
| UYC77766.1 | Putative transmembrane protein | 2.63 |
| UYC76922.1 | CDP-diacylglycerol--glycerol-3-phosphate 3-phosphatidyltransferase (EC 2.7.8.5) | 2.62 |
| UYC77134.1 | DedA protein | 2.61 |
| UYC77594.1 | hypothetical protein | 2.37 |
| UYC78127.1 | hypothetical protein | 2.19 |
| UYC78814.1 | 4-carboxymuconolactone decarboxylase (EC 4.1.1.44) | 2.09 |
| UYC78830.1;UYC76067.1 | Outer membrane low permeability porin, OprD family | 2.07 |
| UYC77224.1 | Alkanesulfonate ABC transporter substrate-binding protein SsuA | 2.06 |
| UYC75575.1 | Bis(5'-nucleosyl)-tetraphosphatase (asymmetrical) (EC 3.6.1.17) | 2.04 |
| UYC78809.1 | 3-oxoadipate CoA-transferase subunit B (EC 2.8.3.6) | 2.01 |
| UYC78708.1 | FIG00350630: hypothetical protein | 2.01 |
| UYC77612.1 | hypothetical protein | -1.99 |
| UYC76332.1 | cupin domain-containing protein | -2.00 |
| UYC77740.1 | probable membrane protein STY1534 | -2.01 |
| UYC79271.1 | sel1 repeat family protein | -2.01 |
| UYC78765.1 | hypothetical protein | -2.02 |
| UYC76464.1 | Phosphoglucomutase (EC 5.4.2.2) @ Phosphomannomutase (EC 5.4.2.8) | -2.03 |
| UYC78171.1 | Transcriptional regulator, LysR family | -2.04 |
| UYC77687.1 | TonB, C-terminal | -2.06 |
| UYC78135.1 | type I-F CRISPR-associated protein Csy3 | -2.06 |
| UYC78409.1 | DUF4760 domain-containing protein | -2.09 |
| UYC76677.1 | hypothetical protein OB946_15875 | -2.11 |
| UYC78968.1;UYC78957.1 | hypothetical protein OB946_09285 | -2.11 |
| UYC75878.1 | Phosphonoacetaldehyde hydrolase (EC 3.11.1.1) | -2.17 |
| UYC76653.1 | Ribosome hibernation promoting factor Hpf | -2.21 |
| UYC77193.1 | hypothetical protein OB946_18720 | -2.22 |
| UYC75967.1 | hypothetical protein OB946_11930 | -2.23 |
| UYC75711.1 | Crossover junction endodeoxyribonuclease RuvC (EC 3.1.22.4) | -2.23 |
| UYC77434.1 | hypothetical protein | -2.23 |
| UYC76495.1 | Carnitine monooxygenase, oxygenase component CntA | -2.24 |
| UYC76155.1 | AAA domain-containing protein | -2.25 |
| UYC76058.1 | Cu(I)-responsive transcriptional regulator | -2.27 |
| UYC77509.1 | hypothetical protein OB946_01300 | -2.27 |
| UYC77414.1 | MBL-fold metallo-hydrolase superfamily | -2.29 |
| UYC75947.1 | hypothetical protein | -2.33 |
| **UYC76454.1** | **Twitching motility protein PilT** | **-2.34** |
| UYC75562.1 | hypothetical protein | -2.36 |
| UYC75922.1 | 3-hydroxyadipyl-CoA dehydrogenase | -2.37 |
| UYC78877.1 | LysR substrate-binding domain-containing protein | -2.38 |
| UYC78632.1 | Periplasmic chorismate mutase I precursor (EC 5.4.99.5) | -2.39 |
| UYC76852.1 | Type III effector HopPmaJ | -2.45 |
| UYC78144.1 | Linoleoyl-CoA desaturase (EC 1.14.19.3) | -2.47 |
| UYC77065.1 | FIGfam050825 | -2.52 |
| UYC78923.1 | DEAD/DEAH box helicase family protein | -2.52 |
| UYC77876.1 | Fatty acid desaturase | -2.53 |
| UYC78598.1 | Acetyltransferase, GNAT family | -2.54 |
| UYC77682.1 | Inner membrane protein YihY, formerly thought to be RNase BN | -2.57 |
| **UYC75925.1** | **1,2-phenylacetyl-CoA epoxidase, subunit E (EC 1.14.13.149)** | **-2.60** |
| UYC76139.1 | Probable coniferyl aldehyde dehydrogenase (EC 1.2.1.68);Aldehyde dehydrogenase (EC 1.2.1.3) | -2.61 |
| UYC78143.1 | Flavodoxin reductases (ferredoxin-NADPH reductases) family 1 | -2.66 |
| UYC78133.1 | type I-F CRISPR-associated protein Csy1 | -2.68 |
| UYC78245.1 | hypothetical protein OB946_05385 | -2.68 |
| UYC75633.1 | ABC transporter ATP-binding protein | -2.71 |
| **UYC75920.1** | **Phenylacetate-coenzyme A ligase (EC 6.2.1.30)** | **-2.74** |
| **UYC77836.1** | **Twitching motility protein PilH** | **-2.74** |
| UYC78134.1 | type I-F CRISPR-associated protein Csy2 | -2.77 |
| UYC76907.1 | Leader peptidase (Prepilin peptidase) (EC 3.4.23.43) / N-methyltransferase (EC 2.1.1.-) | -2.78 |
| **UYC77535.1** | **Type IV pilus biogenesis protein PilQ** | **-2.80** |
| **UYC79020.1** | **Type IV pilus biogenesis protein PilP** | **-2.80** |
| **UYC76455.1** | **Type IV pilus assembly ATPase component PilU** | **-2.81** |
| UYC79102.1 | Histone acetyltransferase HPA2 and related acetyltransferases | -2.84 |
| UYC77374.1 | hypothetical protein OB946_00585 | -2.85 |
| **UYC77557.1** | **Type IV fimbrial biogenesis protein PilX** | **-2.86** |
| UYC77161.1 | D-amino acid dehydrogenase (EC 1.4.99.6) | -2.88 |
| UYC79027.1 | hypothetical protein OB946_02540 | -2.89 |
| UYC77747.1 | hypothetical protein | -2.89 |
| UYC77456.1 | Gamma-aminobutyrate:alpha-ketoglutarate aminotransferase (EC 2.6.1.19) | -2.89 |
| UYC78159.1 | Large repetitive protein | -2.91 |
| **UYC75927.1** | **1,2-phenylacetyl-CoA epoxidase, subunit C (EC 1.14.13.149)** | **-2.93** |
| UYC76816.1 | hypothetical protein | -2.99 |
| **UYC77534.1** | **Type IV pilus biogenesis protein PilO** | **-3.02** |
| **UYC76926.1** | **hypothetical protein OB946_17215** | **-3.07** |
| UYC76152.1 | Oxidoreductase | -3.08 |
| UYC76183.1 | FIG00350110: hypothetical protein | -3.13 |
| UYC77454.1 | gamma-aminobutyrate (GABA) permease | -3.16 |
| UYC76727.1 | FIG00349950: hypothetical protein | -3.18 |
| UYC76608.1 | hypothetical protein | -3.26 |
| UYC75928.1 | 1,2-phenylacetyl-CoA epoxidase, subunit B (EC 1.14.13.149) | -3.27 |
| **UYC77556.1** | **Type IV fimbrial biogenesis protein PilW** | **-3.28** |
| UYC76219.1 | Urease accessory protein UreE | -3.30 |
| **UYC75929.1** | **1,2-phenylacetyl-CoA epoxidase, subunit A (EC 1.14.13.149)** | **-3.32** |
| UYC79070.1 | PEGA domain-containing protein | -3.45 |
| **UYC77533.1** | **Type IV pilus biogenesis protein PilN** | **-3.49** |
| UYC77835.1 | twitching motility protein PilG | -3.75 |
| **UYC77532.1** | **Type IV pilus biogenesis protein PilM** | **-3.76** |
| UYC75753.1 | Tautomerase | -3.90 |
| UYC77005.1 | hypothetical protein | -3.97 |
| UYC75523.1 | Aspartate ammonia-lyase (EC 4.3.1.1) | -4.33 |
| UYC78428.1 | Type II secretory pathway, ATPase PulE/Tfp pilus assembly pathway, ATPase PilB | -4.51 |
| UYC77340.1 | Formiminoglutamase (EC 3.5.3.8) | -4.90 |
| **UYC76906.1** | **Type IV fimbrial assembly protein PilC** | **-5.17** |
| UYC79047.1 | type I-F CRISPR-associated endoribonuclease Cas6/Csy4 | -5.34 |
| UYC77840.1 | hypothetical protein | -5.70 |
| UYC78379.1 | hypothetical protein OB946_06090 | -5.73 |
| UYC78474.1 | Adenosylmethionine-8-amino-7-oxononanoate aminotransferase (EC 2.6.1.62) | -5.74 |
| UYC78424.1 | Uncharacterized protease YegQ | -6.00 |
| UYC76692.1 | Late competence protein ComEA, DNA receptor | -6.04 |
| UYC77025.1 | BRCT domain-containing protein | -7.07 |
| UYC76860.1 | hypothetical protein | -7.28 |
| UYC78168.1 | hypothetical protein | -7.38 |
| UYC78954.1;UYC78945.1 | hypothetical protein OB946_09210 | -7.79 |
| **UYC77837.1** | **Type IV pili signal transduction protein PilI** | **-8.88** |
| **UYC77838.1** | **Type IV pilus biogenesis protein PilJ** | **-9.97** |
| UYC75926.1 | 1,2-phenylacetyl-CoA epoxidase, subunit D (EC 1.14.13.149) | -11.68 |
| **UYC77545.1** | **Type IV pilin PilA** | **-13.07** |
| **UYC77558.1** | **Type IV fimbrial biogenesis protein PilY1** | **-13.25** |
| UYC76460.1 | Hemerythrin domain protein | -15.60 |
| UYC76989.1 | Helix-turn-helix, Fis-type | -30.09 |
| UYC78963.1;UYC78949.1 | zonular occludens toxin domain-containing protein | -31.31 |
| **UYC77839.1** | **Twitching motility protein PilG** | **-32.87** |
|  |  |  |

**a|** Fold change cutoff: 2-fold with a p-value < 0.05. Student’s unpaired *t* test.
