# Supplementary material for: Diclofenac sensitizes multi-drug resistant Acinetobacter baumannii to colistin
Source: PLoS Pathog. 2024 Nov 21;20(11):e1012705. doi: 10.1371/journal.ppat.1012705 (PMC11620633; doi:10.1371/journal.ppat.1012705)
Supplement: S11 Table — (DOCX) [file ppat.1012705.s021.docx]

**Table S11: Differentially expressed proteins in ARC6851 in colistin and diclofenac treatment vs diclofenac.**

| **Accession** | **Annotated protein** | **Fold change^a^** |
| --- | --- | --- |
| UYC75722.1 | Transcriptional regulator, LysR family | 24.51 |
| UYC76950.1 | Protein translocase subunit SecE | 11.54 |
| UYC76459.1 | FIG00351543: hypothetical protein | 7.16 |
| UYC76922.1 | CDP-diacylglycerol--glycerol-3-phosphate 3-phosphatidyltransferase (EC 2.7.8.5) | 6.74 |
| UYC77596.1 | hypothetical protein | 6.59 |
| UYC77191.1 | glycosyltransferase family 4 protein | 4.97 |
| UYC76564.1 | Transcriptional regulator, AcrR family | 4.21 |
| UYC78767.1 | hypothetical protein | 3.90 |
| UYC76504.1 | Multimeric flavodoxin WrbA | 3.17 |
| UYC77594.1 | hypothetical protein | 3.12 |
| UYC77597.1 | hypothetical protein | 3.10 |
| UYC75585.1 | Putative sulfate permease | 3.00 |
| UYC78477.1 | Bsr8028 protein | 2.68 |
| **UYC76566.1** | **Membrane fusion component of MSF-type tripartite multidrug efflux system** | **2.65** |
| UYC76053.1 | FIG00351239: hypothetical protein | 2.49 |
| UYC78867.1 | TetR/AcrR family transcriptional regulator | 2.45 |
| UYC75575.1 | Bis(5'-nucleosyl)-tetraphosphatase (asymmetrical) (EC 3.6.1.17) | 2.44 |
| UYC75736.1 | Glutamate/aspartate ABC transporter, permease protein GltK (TC 3.A.1.3.4) | 2.26 |
| UYC76238.1 | 2-oxoglutarate dehydrogenase complex, dehydrogenase component | 2.24 |
| UYC77860.1 | putative signal peptide;hypothetical protein | 2.22 |
| UYC78817.1 | 3-dehydroshikimate dehydratase (EC 4.2.1.118) | 2.11 |
| UYC76321.1 | hypothetical protein OB946_13920 | 2.06 |
| UYC78878.1 | hypothetical protein | 2.06 |
| **UYC75917.1** | **Acyl-coenzyme A thioesterase PaaD (Pse.pu.) (E. coli PaaI)** | **2.02** |
| UYC77509.1 | hypothetical protein OB946_01300 | -2.01 |
| UYC76914.1 | UPF0301 protein YqgE | -2.05 |
| **UYC76455.1** | **Type IV pilus assembly ATPase component PilU** | **-2.07** |
| **UYC77535.1** | **Type IV pilus biogenesis protein PilQ** | **-2.08** |
| UYC77372.1 | VgrG protein | -2.08 |
| UYC77374.1 | hypothetical protein OB946_00585 | -2.09 |
| UYC76152.1 | Oxidoreductase | -2.10 |
| UYC77005.1 | hypothetical protein | -2.10 |
| UYC76020.1 | hypothetical protein OB946_12265 | -2.12 |
| UYC76727.1 | FIG00349950: hypothetical protein | -2.12 |
| UYC75717.1;UYC78972.1 | Fimbrial protein precursor | -2.16 |
| UYC79027.1 | hypothetical protein OB946_02540 | -2.19 |
| UYC77207.1 | 1,6-anhydro-N-acetylmuramyl-L-alanine amidase | -2.22 |
| UYC76525.1 | LSU ribosomal protein L32p @ LSU ribosomal protein L32p, zinc-independent | -2.24 |
| UYC76495.1 | Carnitine monooxygenase, oxygenase component CntA | -2.27 |
| **UYC77556.1** | **Type IV fimbrial biogenesis protein PilW** | **-2.28** |
| UYC79047.1 | type I-F CRISPR-associated endoribonuclease Cas6/Csy4 | -2.34 |
| **UYC79020.1** | **Type IV pilus biogenesis protein PilP** | **-2.39** |
| UYC76816.1 | hypothetical protein | -2.40 |
| UYC77384.1 | Nucleoside-binding outer membrane protein | -2.43 |
| **UYC77534.1** | **Type IV pilus biogenesis protein PilO** | **-2.47** |
| **UYC77533.1** | **Type IV pilus biogenesis protein PilN** | **-2.48** |
| UYC76450.1 | OsmC/Ohr family protein | -2.51 |
| UYC76183.1 | FIG00350110: hypothetical protein | -2.56 |
| UYC77644.1 | SSU ribosomal protein S19p (S15e) | -2.63 |
| UYC76907.1 | Leader peptidase (Prepilin peptidase) (EC 3.4.23.43) / N-methyltransferase (EC 2.1.1.-) | -2.64 |
| UYC76426.1 | YfdQ family protein | -2.64 |
| **UYC77835.1** | **Twitching motility protein PilG** | **-2.67** |
| UYC77519.1 | Uncharacterized amino acid permease, GabP family | -2.70 |
| UYC76926.1 | hypothetical protein OB946_17215 | -2.73 |
| UYC78424.1 | Uncharacterized protease YegQ | -2.75 |
| UYC78428.1 | Type II secretory pathway, ATPase PulE/Tfp pilus assembly pathway, ATPase PilB | -2.77 |
| UYC76608.1 | hypothetical protein | -2.88 |
| UYC76692.1 | Late competence protein ComEA, DNA receptor | -2.94 |
| UYC75670.1 | MotA/TolQ/ExbB proton channel family protein | -3.01 |
| **UYC77532.1** | **Type IV pilus biogenesis protein PilM** | **-3.03** |
| UYC78382.1;UYC78272.1 | hypothetical protein | -3.20 |
| UYC75750.1 | hypothetical protein | -3.38 |
| UYC78168.1 | hypothetical protein | -3.45 |
| **UYC76906.1** | **Type IV fimbrial assembly protein PilC** | **-3.46** |
| UYC76219.1 | Urease accessory protein UreE | -3.47 |
| UYC76229.1 | Citrate/H+ symporter of CitMHS family | -3.55 |
| UYC75523.1 | Aspartate ammonia-lyase (EC 4.3.1.1) | -4.13 |
| UYC79070.1 | PEGA domain-containing protein | -4.43 |
| UYC77157.1 | D-serine/D-alanine/glycine transporter | -4.54 |
| UYC77025.1 | BRCT domain-containing protein | -4.77 |
| UYC79060.1 | Uncharacterized UPF0033 protein | -4.77 |
| UYC76860.1 | hypothetical protein | -4.96 |
| UYC78954.1;UYC78945.1 | hypothetical protein OB946_09210 | -5.76 |
| **UYC77837.1** | **type IV pili signal transduction protein PilI** | **-5.94** |
| **UYC77838.1** | **Type IV pilus biogenesis protein PilJ** | **-6.04** |
| UYC76488.1 | Protoporphyrinogen IX oxidase, novel form, HemJ (EC 1.3.-.-) | -6.61 |
| **UYC77558.1** | **Type IV fimbrial biogenesis protein PilY1** | **-6.73** |
| UYC76460.1 | Hemerythrin domain protein | -7.16 |
| **UYC77545.1** | **Type IV pilin PilA** | **-9.36** |
| **UYC77839.1** | **Twitching motility protein PilG** | **-17.83** |
| UYC76989.1 | Helix-turn-helix, Fis-type | -18.10 |
| UYC78963.1;UYC78949.1 | zonular occludens toxin domain-containing protein | -23.91 |

**a|** Fold change cutoff: 2-fold with a p-value < 0.05. Student’s unpaired *t* test.
