# Supplementary material for: Diclofenac sensitizes multi-drug resistant Acinetobacter baumannii to colistin
Source: PLoS Pathog. 2024 Nov 21;20(11):e1012705. doi: 10.1371/journal.ppat.1012705 (PMC11620633; doi:10.1371/journal.ppat.1012705)
Supplement: S12 Table — (DOCX) [file ppat.1012705.s022.docx]

**Table S12: Strains used in this study.**

| **Strain** | **Description** | **Source** |
| --- | --- | --- |
| 17978 | *A. baumannii* ATCC 17978 with pAB3 plasmid S/T resistant NCBI accession number CP012004.1 | (1) |
| UPAB1 | Uropathogenic *A. baumannii* clinical isolate | (2) |
| AB347 | *A. baumannii*, respiratory clinical isolate from Bolivia (2016) | (3) |
| AB431 | *A. baumannii*, urinary clinical isolate from United States (2019) | Atlanta |
| AB774 | *A. baumannii*, respiratory clinical isolate from France (2022) | France |
| ARC6851 | *A. baumannii* | (4) |
| ARC6851 ∆*pilA* | ARC6851 *pilA* mutant | This study |
| AR0129 | *K. pneumoniae* | CDC Panel Name Enterobacterales Carbapenemase Diversity (CRE) AR Bank Number 0129 |
| AR0046 | *K. pneumoniae* | CDC Panel Gram Negative Carbapenemase Detection (CarbaNP) AR Bank Number 0046 |
| AR0126 | *K. pneumoniae* | CDC Panel Name Enterobacterales Carbapenemase Diversity (CRE) AR Bank Number 0126 |
| KR49 | *K. pneumoniae* | CDC KR Bank Number 49 |
| AR0125 | *K. pneumoniae* | CDC Panel Name Enterobacterales Carbapenemase Diversity (CRE) AR Bank Number 0125 |
| AR0073 | *E. cloacae* | CDC Panel Gram Negative Carbapenemase Detection (CarbaNP) AR Bank Number 0073 |
| 409957 | *P. aeruginosa* | (5) |
| 369569 | *P. aeruginosa* | (5) |
| 358800 | *P. aeruginosa* | (5) |
| *S. aureus* | *S. aureus* | Newman |

**SUPPLEMENTARY REFERENCES**

1. Weber BS, Ly PM, Irwin JN, Pukatzki S, Feldman MF. A multidrug resistance plasmid contains the molecular switch for type VI secretion in Acinetobacter baumannii. Proc Natl Acad Sci U S A. 2015;112(30):9442-7.

2. Di Venanzio G F-mA, Calix JJ, Haurat MF, Scott NE, Palmer LD, Potter RF, Hibbing ME, Friedman L, Wang B, Dantas G, Skaar EP, Hultgren SJ, Feldman MF. Urinary tract colonization is enhanced by a plasmid that regulates uropathogenic Acinetobacter baumannii chromosomal genes. Nat Commun 2019;10:1–13.

3. Cerezales M, Xanthopoulou K, Wille J, Bustamante Z, Seifert H, Gallego L, et al. Acinetobacter baumannii analysis by core genome multi-locus sequence typing in two hospitals in Bolivia: endemicity of international clone 7 isolates (CC25). Int J Antimicrob Agents. 2019;53(6):844-9.

4. McGuffey JC, Jackson-Litteken CD, Di Venanzio G, Zimmer AA, Lewis JM, Distel JS, et al. The tRNA methyltransferase TrmB is critical for Acinetobacter baumannii stress responses and pulmonary infection. mBio. 2023:e0141623.

5. Lebreton F, Snesrud E, Hall L, Mills E, Galac M, Stam J, et al. A panel of diverse Pseudomonas aeruginosa clinical isolates for research and development. JAC Antimicrob Resist. 2021;3(4):dlab179.
