# Supplementary material for: Diclofenac sensitizes multi-drug resistant Acinetobacter baumannii to colistin
Source: PLoS Pathog. 2024 Nov 21;20(11):e1012705. doi: 10.1371/journal.ppat.1012705 (PMC11620633; doi:10.1371/journal.ppat.1012705)
Supplement: S13 Table — (DOCX) [file ppat.1012705.s023.docx]

**Table S13: Plasmids used in this study.**

| **Plasmid** | **Description^a^** | **Source** |
| --- | --- | --- |
| pKD4-Apr | Source for Apramycin cassette for mutant generation, Apr^r^ | (1) |
| pAT03 | pMMB67EH with FLP recombinase | (2) |
| pAT04 | pMMB67EH with RecAbsystem, Hyg | (2) |
| pUCT18T-miniTn7T-Zeo | mTn7 complementation vector, Zeo^r^ | (3) |
| pUCT18T-miniTn7T-Zeo-ARC_*pilA* | ARC6851 Δ*pilA* complementation construct, Zeo^r^ | This study |

^a^Apr, apramycin; Zeo, zeocin

**SUPPLEMENTARY REFERENCES**

1. McGuffey JC, Jackson-Litteken CD, Di Venanzio G, Zimmer AA, Lewis JM, Distel JS, et al. The tRNA methyltransferase TrmB is critical for Acinetobacter baumannii stress responses and pulmonary infection. mBio. 2023:e0141623.

2. Tucker AT, Nowicki EM, Boll JM, Knauf GA, Burdis NC, Trent MS, et al. Defining gene-phenotype relationships in Acinetobacter baumannii through one-step chromosomal gene inactivation. mBio. 2014;5(4):e01313-14.

3. Ducas-Mowchun K, De Silva PM, Crisostomo L, Fernando DM, Chao TC, Pelka P, et al. Next Generation of Tn7-Based Single-Copy Insertion Elements for Use in Multi- and Pan-Drug-Resistant Strains of Acinetobacter baumannii. Appl Environ Microbiol. 2019;85(11).
