# Supplementary material for: Diclofenac sensitizes multi-drug resistant Acinetobacter baumannii to colistin
Source: PLoS Pathog. 2024 Nov 21;20(11):e1012705. doi: 10.1371/journal.ppat.1012705 (PMC11620633; doi:10.1371/journal.ppat.1012705)
Supplement: S14 Table — (DOCX) [file ppat.1012705.s024.docx]

**Table S14: Primers used in this study**

| **Primers** | **Sequence** |
| --- | --- |
| F_U_*pilA*_1000bp_KO | TGCTGGCGTAGTAATGAG |
| R_U_*pilA*_1000bp_KO | CCAGCCTACACAATCGCTATTCATAGCCTTTTCCCC |
| F_D_*pilA*_1000bp_KO | AAGGAGGATATTCATATGTTCTGCTTGCCCTGC |
| R_D_*pilA*_1000bp_KO | CCACATCCCTATACGC |
| F_prom(500bp)_pilA_puCT18T-Zeo | GAGAAGCTTGGGCCCGGTACCGTAAGTCGATTGTAGAGCAGC |
| R_prom(500bp)_pilA_puCT18T-Zeo | GCAAGGCCTTCGCGAGGTACCTTATGCTGCAGGGCAAC |
| F_*pilA*_ARC6851_qRT-PCR | ACTCATGATCGTAGTTGCCATT |
| R_*pilA*_ARC6851_qRT-PCR | TTCACTAACCGCACGTGATAC |
| F_*pilA*_347_qRT-PCR | TGGTTGCCATTATCGGTATCT T |
| R_*pilA*_347_qRT-PCR | GGACAGCTGCTGCTACATTA |
| F_*rpoB*_qRT-PCR | ACGGTACTGAGCGTGTAATC |
| R_*rpoB*_qRT-PCR | TTACCACTTGAGTGGGTCTTAC |
