# Supplementary material for: Diclofenac sensitizes multi-drug resistant Acinetobacter baumannii to colistin
Source: PLoS Pathog. 2024 Nov 21;20(11):e1012705. doi: 10.1371/journal.ppat.1012705 (PMC11620633; doi:10.1371/journal.ppat.1012705)
Supplement: S1 Appendix — (DOCX) [file ppat.1012705.s025.docx]

**SUPPLEMENTARY MATERIAL AND METHODS**

**Antibiotic growth curves:** Growth curves were performed in sterile, round-bottom, polystyrene, 96-well plates (Corning 3788). Bacterial cultures were started from LB agar plates, inoculated in LB broth, and grown 16 h at 37°C and 200 rpm. Overnight cultures were subcultured for 3 hours and diluted in fresh medium to an OD_600_ of 0.01 and inoculated into 96-well plates at a final volume of 150 μL with or without colistin and/or DMSO/diclofenac. Plates were incubated at 37°C in shaking conditions for 16h in a BioTek microplate reader, with OD_600_ values measured at 30 minutes intervals. When indicated, colistin (1 µg/ml, 3 µg/ml, 256 µg/ml), diclofenac (25 µM, 40 µM, 50 µM, 60 µM, 75 µM, 100 µM) vancomycin (8 µg/ml ,16 µg/ml, 32 µg/ml) SDS (0.01%), EDTA (0.075 mM) were added. All experiments were performed on at least 3 independent days with at least 3 technical replicates per strain per condition.
**Construction of *pilA* mutant in ARC6851:** The primers used in this study are listed in **S14 Table.** ARC6851 deletion mutant in *pilA* was constructed as previously described (1). Briefly, 1kb upstream and downstream of *pilA* gene were amplified from ARC6851 genomic DNA. Overlap extension PCR fused the fragments with an FRT site-flanked apramycin resistance cassette that was amplified from an apramycin variant of pKD4 (2). The purified linear DNA product was electroporated into ARC6851 wild-type strain containing the pAT04 plasmid that encodes an IPTG-inducible copy of RecAB recombinase, and mutants were selected using resistance to apramycin treatment (1). To excise the antibiotic cassette, a plasmid encoding an IPTG-inducible copy of FLP recombinase, PAT03, was transformed into ARC6851 lacking *pilA* (1). Clean mutants were confirmed via return of apramycin sensitivity and whole-genome sequencing.

**Functional analysis of RNA sequencing results:** To functionally annotate genes in our RNA sequencing data sets, we used eggNOG Mapper (3, 4). FASTA sequences were uploaded to the eggNOG Mapper website. Genes were categorized based on clusters of orthologous groups (COG). COG functions were then organized into pie charts using GraphPad Prism.

**Transmission electron microscopy**: ARC6851 was grown overnight in LB before being diluted into 10mL of LB, LB plus diclofenac (0.1 mM), LB plus colistin (1 μg/ml), or LB plus diclofenac and colistin and grown for 2 h at 37°C with shaking. Next, cultures were washed with PBS and used for TEM. For negative staining and analysis by TEM, bacterial samples were fixed with 1% glutaraldehyde (Ted Pella) and allowed to absorb onto freshly glow discharged Formvar/carbon-coated copper grids for 10 minutes. Grids were then washed in dH_2_O and stained with 1% aqueous uranyl acetate (Ted Pella) for 1 minute. Excess liquid was gently wicked off, and grids were allowed to air dry. Samples were viewed on a JEOL 1200EX transmission electron microscope equipped with an AMT 8-megapixel digital camera (Advanced Microscopy Techniques). Total pili was quantified in twenty bacteria using 12000X magnification in DMSO and LB plus diclofenac and colistin.

**qRT-PCR***: A. baumannii* ARC6851 and AB347 were grown overnight in LB before being diluted into 10mL of LB, LB plus diclofenac (100 µM), LB plus colistin (1 μg/ml), or LB plus diclofenac and colistin and grown for 2 h at 37°C with shaking. TRIzol was added and the samples were flash frozen. RNA was extracted from thawed samples using a chloroform extraction in conjunction with the Qiagen RNeasy Mini Kit. To remove contaminating DNA, both the Qiagen on-column DNase treatment and an off-column rigorous DNase treatment using the TURBO DNA-*free* kit were used. For reverse transcription (RT)-PCR, cDNA was prepared from 1 µg RNA using a high-capacity RNA-to-cDNA kit (Applied Biosystems), according to the manufacturer’s protocol. The cDNA was diluted to 10 ng/µL, and 1 µL was used as template for quantitative PCR (qPCR) using PowerUp SYBR green master mix (Applied Biosciences) on a ViiA7 real-time PCR machine (Applied Biosystems), following the manufacturer’s suggested protocol. The *A. baumannii rpoB* gene were used as the reference gene. All primers used for qPCR were designed using IDT PrimerQuest and are listed in **S14 Table**. Threshold cycle (*C_T_*) values were normalized to the average of *rpoB*, and fold changes and Log_2_(fold changes) were calculated using the ΔΔ*C_T_*method.

**Determination of cyclooxygenase activity:** Lung lysates containing protease inhibitors were used to determine COX-2 activities using a COX fluorescent activity assay kit (Cayman Chemical, #700200, Ann Arbor, MI, USA). The assay utilizes the peroxidase component of COXs and the production of resorufin. Resorufin fluorescence can be analyzed with an excitation wavelength between 530-540 nm and an emission wavelength between 585-595 nm.

**SUPPLEMENTARY REFERENCES**

1. Tucker AT, Nowicki EM, Boll JM, Knauf GA, Burdis NC, Trent MS, et al. Defining gene-phenotype relationships in Acinetobacter baumannii through one-step chromosomal gene inactivation. mBio. 2014;5(4):e01313-14.

2. Datsenko KA, Wanner BL. One-step inactivation of chromosomal genes in Escherichia coli K-12 using PCR products. Proc Natl Acad Sci U S A. 2000;97(12):6640-5.

3. Cantalapiedra CP, Hernandez-Plaza A, Letunic I, Bork P, Huerta-Cepas J. eggNOG-mapper v2: Functional Annotation, Orthology Assignments, and Domain Prediction at the Metagenomic Scale. Mol Biol Evol. 2021;38(12):5825-9.

4. Huerta-Cepas J, Szklarczyk D, Heller D, Hernandez-Plaza A, Forslund SK, Cook H, et al. eggNOG 5.0: a hierarchical, functionally and phylogenetically annotated orthology resource based on 5090 organisms and 2502 viruses. Nucleic Acids Res. 2019;47(D1):D309-D14.
