# Supplementary material for: Diclofenac sensitizes multi-drug resistant Acinetobacter baumannii to colistin
Source: PLoS Pathog. 2024 Nov 21;20(11):e1012705. doi: 10.1371/journal.ppat.1012705 (PMC11620633; doi:10.1371/journal.ppat.1012705)
Supplement: Attachment — Submitted filename: Review upload.docx [file ppat.1012705.s026.docx]

This manuscript describes the discovery of synergies between colistin and diclofenac (DF) on killing or inhibiting the growth of colistin-resistant *Acinetobacter baumannii* strains *in vitro* and *in vivo* in a murine model of acute pneumonia. This synergistic effect was observed first with *A. baumannii* isolates *in vitro* and extended to other bacteria including *Klebsiella pneumoniae, Enterobacter cloacae* and *Pseudomonas aeruginosa.* Studies with *A. baumannii* in the mouse model indicated similar synergistic effect of colistin and DF in reducing mortality as well as bacterial loads in lungs, kidneys and spleens. The mechanisms of this synergy were investigated by transcriptomics and proteomics, with the results suggesting a role for the bacterial type IV pilus (T4P). The *pilA* gene encodes the pilin for T4P. Interestingly, a *pilA* mutant, which is as virulent in the mouse model as the wild type, still displayed a synergistic response to colistin and DF *in vitro*. Yet, in the mouse model, colistin alone reduced the bacterial loads of the *pilA* mutant without DF. The authors suggested that the mechanisms of synergy are possibly related to the negative effect of DF on T4P formation, which in turn reduces biofilm formation or sequestration of colistin by the post-translationally glycosylated pilins.

The manuscript is well written, and the work clearly described. The discovery of synergy between colistin and DF could have significant clinical implications in the treatment of colistin-resistant infections in a variety of bacteria. This is also the first example of a potential anti-T4P compound with antivirulence properties in a bacterium other than neisserial species.

I have a few minor comments on the manuscripts listed below.

1. The data on virulence by the *pilA* mutant is not shown, neither is data on the effect of treatments with colistin and/or DF on mouse survival. These could be provided as part of or as supplemental to Fig. 8.
2. The horizontal lines (for median) and error bars in Figs. 6, 7 & 8 appeared misplaced or missing at times. This could be a file conversion issue as well.
3. Double check Figure legends to ensure clarity and readability.
4. While the mechanism of synergy is possibly related to T4P in *A. baumannii*, other bacteria such as *K. pneumoniae* are not known to have T4P. This point may be covered in Discussion.
