# Supplementary material for: Diclofenac sensitizes multi-drug resistant Acinetobacter baumannii to colistin
Source: PLoS Pathog. 2024 Nov 21;20(11):e1012705. doi: 10.1371/journal.ppat.1012705 (PMC11620633; doi:10.1371/journal.ppat.1012705)
Supplement: Attachment — Submitted filename: Response_to_reviewers_cover_letter.docx [file ppat.1012705.s027.docx]

Washington University in St. Louis School of Medicine

Department of Molecular Microbiology,

660 S. Euclid Avenue, MSC-8230-16-09,

Saint Louis, Missouri 63110-1093

October 17, 2024

Dear Editor,

Please find enclosed the revised version of our manuscript entitled “Diclofenac sensitizes multi-drug resistant *Acinetobacter baumannii* to colistin” by Bisaro *et al*.

We would like to thank the reviewers for their positive comments. We have addressed their concerns and added new data throughout the manuscript. Directly below this letter, you will find our detailed responses to the reviewers’ concerns.

We hope you will find this revised version suitable for publication in *PlosPathogens.*

Sincerely,

Mario F. Feldman, Ph.D.

Professor of Molecular Microbiology

Washington University School of Medicine

Department of Molecular Microbiology

[mfeldman@wustl.edu](mailto:mfeldman@wustl.edu)

Tel. 314-747-4473

Your manuscript has been reviewed by two experts and I would now like you to revise your study in line with their feedback. Both reviewers recognized the potentially high translational impact of the study, however they also highlighted the need for further exploration of mechanisms/modes of action underlying the observed interaction between diclofenac and colistin. They also requested clarification on the mechanism(s) of colistin resistance in the strains used in the study.

**Reviewer #1** uploaded their comments as an attachment, and they are copied over here:
This manuscript describes the discovery of synergies between colistin and diclofenac (DF) on killing or inhibiting the growth of colistin- resistant Acinetobacter baumannii strains in vitro and in vivo in a murine model of acute pneumonia. This synergistic effect was observed first with A. baumannii isolates in vitro and extended to other bacteria including Klebsiella pneumoniae, Enterobacter cloacae and Pseudomonas aeruginosa. Studies with A. baumannii in the mouse model indicated similar synergistic effect of colistin and DF in reducing mortality as well as bacterial loads in lungs, kidneys and spleens. The mechanisms of this synergy were investigated by transcriptomics and proteomics, with the results suggesting a role for the bacterial type IV pilus (T4P). The pilA gene encodes the pilin for T4P. Interestingly, a pilA mutant, which is as virulent in the mouse model as the wild type, still displayed a synergistic response to colistin and DF in vitro. Yet, in the mouse model, colistin alone reduced the bacterial loads of the pilA mutant without DF. The authors suggested that the mechanisms of synergy are possibly related to the negative effect of DF on T4P formation, which in turn reduces biofilm formation or sequestration of colistin by the post-translationally glycosylated pilins.
The manuscript is well written, and the work clearly described. The discovery of synergy between colistin and DF could have significant clinical implications in the treatment of colistin-resistant infections in a variety of bacteria. This is also the first example of a potential anti-T4P compound with antivirulence properties in a bacterium other than neisserial species.
I have a few minor comments on the manuscripts listed below.
1. The data on virulence by the pilA mutant is not shown, neither is data on the effect of treatments with colistin and/or DF on mouse survival. These could be provided as part of or as supplemental to Fig. 8.

We would like to thank the reviewer for identifying this oversight. Survival data is presented in **Fig 7B** and the bacterial burden from the *pilA* mutants strains is shown in the revised **Fig. S10**.

2. The horizontal lines (for median) and error bars in Figs. 6, 7 & 8 appeared misplaced or missing at times. This could be a file conversion issue as well.

We thank the reviewer for the comment. We have reviewed the original Prism files, and they are consistent with the files presented in the manuscript. **Fig. 6** shows the Median while **Fig 7, 8** and **S10** show the Mean with SEM.
3. Double check Figure legends to ensure clarity and readability.

We appreciate the reviewer’s observation. We edited the figure legends to enhance clarity and readability.
4. While the mechanism of synergy is possibly related to T4P in A. baumannii, other bacteria such as K. pneumoniae are not known to have T4P. This point may be covered in Discussion.

We thank the reviewer for the comment, a paragraph was added to the discussion to address this (lines 352-366).

Reviewer's Responses to Questions

**Part I - Summary**

Please use this section to discuss strengths/weaknesses of study, novelty/significance, general execution and scholarship.

Reviewer #1: (No Response)

Reviewer #2: In this work, Bisaro and coworkers explore the therapeutic potential of diclofenac, a widely used non-steroidal anti- inflamamtory drug, againt colistin resistant of A, baumannii. Whereas diclofeniac is not toxic, combination wityh colistin exerts a toxic effect against A. baumannii, and also Pseudomonas, Enterobacter, and Klebsiella. Transcriuptomic and proteomic analysis revealed that diclofenic and colistin result in upregualtion of ROS-related systtems and downregualtion of type IV pili. In vivo work probing an intranasal pneumonia model, unveiled the efficacy of combining both drugs.

General comment.
It is urgent to develop new therapeutics against infections such as those caused by A. baumannii. The idea of repurposing drugs is not novel but there is limited evidence in the case of acute infections demonstrating that it is worth exploring in clinical trials. As such this work is timeliness with translational promise. The work design and technical approach is sensible and the experiments include the relevant controls. The in vivo work is perfectly executed. Nonetheless, it remains an open question the mechanism of action of the drugs. I hope authors will find my comments useful to strengthen this important work.

We thank the reviewer for their time and the constructive comments. We agree in the urgency to develop new therapeutics and believe that by repurposing previously studied drugs we can meet the urgent need for novel treatments.

**Part II – Major Issues: Key Experiments Required for Acceptance**

https://www.editorialmanager.com/ppathogens/ViewLetter.aspx?id=2615900&lsid={A3BB9B3A-7438-4A57-8FBA-1B73D8363DF3} Page 2 of 4

View Letter 8/26/24, 10:43 AM

Please use this section to detail the key new experiments or modifications of existing experiments that should be absolutely required to validate study conclusions.

Generally, there should be no more than 3 such required experiments or major modifications for a "Major Revision" recommendation. If more than 3 experiments are necessary to validate the study conclusions, then you are encouraged to recommend "Reject".

Reviewer #1: (No Response)

Reviewer #2: 1. At least n the case of A. baumannii authors need to provide evidence of the mechanisms of colistin resistance of the strains. This will This information should be complemented with the MIC to other antibiotics. It will be also interesting if the authors could also confirm the mechanisms of resistant in Klebsiella and Pseudomonas. To help in the understanding of the system, authors should demonstrate the mechanism of resistance of strain ARC6851.

We thank the reviewer for their insightful observation. Resistance mechanisms to colistin have been already described in *A. baumannii* and are detailed in lines 86-93, in the introduction of the manuscript. MICs to other antibiotics are shown in table S4. Further details had been added throughout the manuscript.

Regarding the reviewer comment, ARC6851 encodes several genes related to resistance to colistin: *pmrC* (OB946_RS03455) that mediates the addition of phosphoetanolamine moieties to lipid A. *pmrC* is upstream of the response regulator transcription factor *pmrA* (OB946_RS03460) and the sensor histidine kinase *pmrB* (OB946_RS03465). Moreover, ARC6851 encodes the EmrAB efflux system, including *emrA* (OB946_RS14570) and *emrB* (OB946_RS14565). This information is now included in the manuscript (lines 117-119).

Regarding *Klebsiella* and *Pseudomonas*, we do not have the sequences of these genomes. Our work is not focused on these bacterial species. During the revision our manuscript, another group reported synergy between diclofenac and colistin against *Klebsiella* and *Pseudomonas* (Fu Q, Liu S, Hu P, Chen H, Zheng J, Shi S, Xu M, Zhou T, Sun Y. Diclofenac Sodium Restores the Sensitivity of Colistin-Resistant Gram-Negative Bacteria to Colistin. ACS Infect Dis. 2024 Aug 9;10(8):2860-2869. doi: 10.1021/acsinfecdis.4c00207. Epub 2024 Jul 29. PMID: 39073350). In the future we will also work in these pathogens to determine if the mechanism of action of the drugs is the same in *A. baumannii*, *Klebsiella* and *Pseudomonas*.

2. The permeability-based experiments could be improved by adding uncouplers because in the current set up authors cannot differentiate membrane permeability versus efflux pumps. Also, authors may consider using as a probe NPN; it does reflect more accurately membrane permeability than the dye used.

As suggested by the reviewer we measured membrane permeabilization with NPN and in the presence the efflux pump inhibitor CCCP. Addition of CCCP did not influence cell permeability in any condition. This figure is now included in the paper as Supplementary **Fig. S2B**.

3. A useful control experiment is to test whether diclofenac increases the susceptibility to vancomycin.

As per suggestion of the reviewer we performed growth curves with vancomycin and diclofenac and we found that diclofenac did not increased the susceptibility to vancomycin.

4. To probe (or not) the membrane effect, authors should test whether diclofenac increases the interaction of colistin/polymyxin B with the surface of Acinetobacter. There are commercially available labeled compounds that can be used for these experiments.

We thank the reviewer for this suggestion. However, we believe that, in agreement with other reports, Our data, along with the new experiments included in the revised manuscript, clearly demonstrate that colistin increases membrane permeability. On the contrary, **Fig. 3A** and **S2** clearly shows that diclofenac does not affect membrane permeability in *Acinetobacter*.

5. To further assess the membrane effect, authors should consider testing the combination of polymyxin B nanopetide and diclofenac. The nonapeptide is normally excluded (although it does interact with the LPS and disorganizes the membrane) but if the membrane affected then it can enter the cell and kill Gram-negative pathogens. The nano peptide also increases membrane permeability.

As per suggestion of the reviewer we performed growth curves with polymyxin B Nonapeptide (PBNP) and diclofenac and we found no significant growth differences in the presence of DF, further supporting our conclusion that the effect of colistin is necessary for the synergistic effect with diclofenac This result is included as **Fig. S3A**.

6. Authors can test whether the mechanism of action is ROS-dependent by testing ROS inhibitors. While intriguing, the results show in Fig 6 only indicate additive effect of ROS not a mechanistic connection between the drugs and their mode of action.

We thank the reviewer for this suggestion. Mi H. et al., 2016 reported that the ROS scavenging activity of DMSO reduces rapid antimicrobial-mediated killing in *E. coli*. We show now that addition of up to 1% DMSO improves ARC6851 growth in presence of DF and colistin. Furthermore, during the revision of this manuscript, Qingxia Fu et al., 2024 (DOI: 10.1021/acsinfecdis.4c00207) reported that ROS production under colistin plus diclofenac treatment, was found to be significantly increased compared to both the monotherapy and blank treatment groups. This information has been added to the manuscript in lines (237-239) and **Fig. 6B**. This result supports the model that DF and colistin effects are mediated by ROS.

7. Is it possible that the effect on type IV pili is ROS-dependent? This can be addressed experimentally.

We thank the reviewer for the insightful comment. Although this is a very interesting hypothesis, the proposed experiment falls outside the scope of the current manuscript and is planned for future research.

8. If the decrease in type IV pili is part of the mechanisms of action then the following can be anticipated: (i) in the strain background tested by the authors, a type IV pili may already display a decrease in the MIC for colistin; and.or (ii) treating this mutant with diclofenac should be toxic already, and/or (iii) treating the mutant with colistin may replace the need for diclofenac. In these experiments, authors should also test the role of ROS as indicated before.

**Fig. 8** and **S9** shows that i) and ii) do not happen *in vitro* (**Fig. S9**), but that *in vivo*, treatment with colistin alone was sufficient to reduce the burden of the Δ*pilA* strain to a similar extent as observed with colistin plus diclofenac treatment (**Fig. 8**)**.** We believe this is due to an anti-virulence effect related to a possible role of type IV pili in pathogenesis. This is discussed in lines (361-366).

9. Authors need to justify why they only tested female mice. It is not longer appropriate to exclude one sex in infection experiments. This reviewer does not ask to repeat the in vivo work testing mal mice but urges the authors to consider this for future work. Additionally, this should be included in the disucssion as a limitation.

We appreciate the reviewer’s insightful comment. Sílvia Pires et al., 2020 studied how “Biological sex influences susceptibility to *Acinetobacter baumannii* pneumonia in mice”. Through their studies using a murine model of acute pneumonia with *A*. *baumannii*, the authors observed that female mice were more susceptible to infection. We added this information as a limitation of our study (lines 426-428).

**Part III – Minor Issues: Editorial and Data Presentation Modifications**

Please use this section for editorial suggestions as well as relatively minor modifications of existing data that would enhance clarity.

Reviewer #1: (No Response)

Reviewer #2:

1. Authors may wish to show in the firsts section of the manuscript the fact that the results are colistin specific (now line 167 of the results).

We thank the reviewer for the suggestion, however we believe that the organization of the result section is clear as it is.
2. Authors may wish to include a cartoon depicting the main findings and the mechanism of action.

We thank the reviewer for the suggestion. We have included a final figure (**Fig. 9**) with our main findings.
3. Please do include the relevant ethics permission reference for the animal work.

All animal experiments were approved by the Washington University Animal Care and Use Committee, and we have complied with all relevant ethical regulations.

PLOS authors have the option to publish the peer review history of their article (what does this mean?). If published, this will include your full peer review and any attached files.

If you choose “no”, your identity will remain anonymous but your review may still be made public.
**Do you want your identity to be public for this peer review?** For information about this choice, including consent withdrawal,

please see our Privacy Policy. Reviewer #1: No
Reviewer #2: No

Figure Files:

While revising your submission, please upload your figure files to the Preflight Analysis and Conversion Engine (PACE) digital diagnostic tool, https://pacev2.apexcovantage.com. PACE helps ensure that figures meet PLOS requirements. To use PACE, you must first register as a user. Then, login and navigate to the UPLOAD tab, where you will find detailed instructions on how to use the tool. If you encounter any issues or have any questions when using PACE, please email us at figures@plos.org.

Data Requirements:

https://www.editorialmanager.com/ppathogens/ViewLetter.aspx?id=2615900&lsid={A3BB9B3A-7438-4A57-8FBA-1B73D8363DF3} Page 3 of 4

View Letter 8/26/24, 10:43 AM

Please note that, as a condition of publication, PLOS' data policy requires that you make available all data used to draw the conclusions outlined in your manuscript. Data must be deposited in an appropriate repository, included within the body of the manuscript, or uploaded as supporting information. This includes all numerical values that were used to generate graphs, histograms etc.. For an example see here on PLOS Biology: http://www.plosbiology.org/article/info%3Adoi%2F10.1371%2Fjournal.pbio.1001908#s5.

Reproducibility:

To enhance the reproducibility of your results, we recommend that you deposit your laboratory protocols in protocols.io, where a protocol can be assigned its own identifier (DOI) such that it can be cited independently in the future. Additionally, PLOS ONE offers an option to publish peer-reviewed clinical study protocols. Read more information on sharing protocols at https://plos.org/protocols? utm_medium=editorial-email&utm_source=authorletters&utm_campaign=protocols

*In compliance with data protection regulations, you may request that we remove your personal registration details at any time. (Remove my information/details). Please contact the publication office if you have any questions.*
